# A subset of cerebrovascular pericytes originates from mature macrophages in the very early phase of vascular development in CNS

**DOI:** 10.1038/s41598-017-03994-1

**Published:** 2017-06-20

**Authors:** Seiji Yamamoto, Masashi Muramatsu, Erika Azuma, Masashi Ikutani, Yoshinori Nagai, Hiroshi Sagara, Bon-Nyeo Koo, Satomi Kita, Erin O’Donnell, Tsuyoshi Osawa, Hiroyuki Takahashi, Ken-ichi Takano, Mitsuko Dohmoto, Michiya Sugimori, Isao Usui, Yasuhide Watanabe, Noboru Hatakeyama, Takahiro Iwamoto, Issei Komuro, Kiyoshi Takatsu, Kazuyuki Tobe, Shumpei Niida, Naoyuki Matsuda, Masabumi Shibuya, Masakiyo Sasahara

**Affiliations:** 1 Department of Pathology, University of Toyama, Toyama, Japan; 2 Institute of Resource Development and Analysis, Kumamoto University, Kumamoto, Japan; 3 Department of Technology Development, Astellas Pharma Tech Co., Ltd., Toyama, Japan; 4 Department of Immunobiology and Pharmacological Genetics, University of Toyama, Toyama, Japan; 5 Medical Proteomics Laboratory, Institute of Medical Science, University of Tokyo, Tokyo, Japan; 6 Department of Anesthesiology, Yonsei University College of Medicine, Seoul, Korea; 7 Laboratory of Stem Cell and Neuro-Vascular Biology, Genetics and Developmental Biology Center, National Heart, Lung, and Blood Institute, National Institutes of Health, Maryland, USA; 8 Department of Pharmacology, Faculty of Medicine, Fukuoka University, Fukuoka, Japan; 9 Laboratry for Systems Biology and Medicine, Research Center for Advanced Science and Technology, The University of Tokyo, Tokyo, Japan; 10 Division for Health Service Promotion, The University of Tokyo, Tokyo, Japan; 11 Departments of Pharmacology, Weill Cornell Medical College, New York, USA; 12 Genome Biotechnology Laboratory, Kanazawa Institute of Technology, Ishikawa, Japan; 13 Department of Integrative Neuroscience, University of Toyama, Toyama, Japan; 14 First Department of Internal Medicine, University of Toyama, Toyama, Japan; 15 Faculty of Medicine, School of Nursing, Hamamatsu University School of Medicine, Shizuoka, Japan; 16 Department of Anesthesiology, Graduate School of Medicine, Aichi Medical University, Aichi, Japan; 17 Department of Cardiovascular Medicine, The University of Tokyo Graduate School of Medicine, Tokyo, Japan; 18 Toyama Prefectural Institute for Pharmaceutical Research, Toyama, Japan; 19 Medical Genome Center, Center for Geriatrics and Gerontology, Aichi, Japan; 20 Department of Emergency and Critical Care Medicine, Nagoya University, Nagoya, Japan; 21 Department of Research and Education, Jobu University, Gunma, Japan; 22 JST, PRESTO, Kawaguchi, Saitama, Japan

## Abstract

Pericytes are believed to originate from either mesenchymal or neural crest cells. It has recently been reported that pericytes play important roles in the central nervous system (CNS) by regulating blood-brain barrier homeostasis and blood flow at the capillary level. However, the origin of CNS microvascular pericytes and the mechanism of their recruitment remain unknown. Here, we show a new source of cerebrovascular pericytes during neurogenesis. In the CNS of embryonic day 10.5 mouse embryos, CD31^+^F4/80^+^ hematopoietic lineage cells were observed in the avascular region around the dorsal midline of the developing midbrain. These cells expressed additional macrophage markers such as CD206 and CD11b. Moreover, the CD31^+^F4/80^+^ cells phagocytosed apoptotic cells as functionally matured macrophages, adhered to the newly formed subventricular vascular plexus, and then divided into daughter cells. Eventually, these CD31^+^F4/80^+^ cells transdifferentiated into NG2/PDGFRβ/desmin-expressing cerebrovascular pericytes, enwrapping and associating with vascular endothelial cells. These data indicate that a subset of cerebrovascular pericytes derive from mature macrophages in the very early phase of CNS vascular development, which in turn are recruited from sites of embryonic hematopoiesis such as the yolk sac by way of blood flow.

## Introduction

It is largely accepted that cerebrovascular pericytes enwrap cerebral blood vessels through their foot processes^1–3^. In addition, it was recently reported that pericytes play an important role in the regulation of blood flow in the brain at the capillary level^4, 5^. Pericytes are also important for blood-brain barrier (BBB) stability^6–8^. Insufficient cerebrovascular pericyte recruitment has been reported in mice lacking platelet-derived growth factor-B (PDGF-B) or platelet-derived growth factor receptor beta (PDGFRβ)^9, 10^. Such deficiencies lead to endothelial hyperplasia, impaired endothelial differentiation, increased vascular leakage, and the formation of rupturing microaneurysms. Mice carrying mutated PDGF-B or with conditionally regulated endothelium-specific PDGF-B expression have a hypomorphic pericyte phenotype. These mice show increased water content in their brains resulting from BBB perturbations such as excess endothelial transcytosis and altered astrocyte end-foot polarization^6^. In the embryonic phase, pericytes also play a critical role in BBB function. More than a week before astrocyte generation, pericyte-endothelial cell interactions are crucial for the regulation of BBB formation, and disruption of these interactions leads to BBB dysfunction^7^. In a previous report, we clearly demonstrated that the pericyte recruitment disorder in a mouse with postnatally-induced systemic depletion of PDGFRβ shows BBB disruption and severe vascular leakage after stroke induced by photothrombotic middle cerebral artery occlusion^11^.

Several lines of experimental evidence have suggested that macrophage subsets contribute to vascular development in both physiological and pathological conditions. In the developing mouse brain, macrophages act as cellular chaperones for vascular anastomosis^12^. These macrophages share molecular similarities with the pro-angiogenic tissue macrophages that are important for vascular development. In the developing retina, myeloid cells control retinal vascular density^13^. These cells contribute to normal development of the retinal vasculature depending on the non-canonical Wnt-Flt1 pathway. In pathological conditions, macrophage subsets contribute to atheroma development in atherosclerosis, which is a major cause of death worldwide^14^. In other conditions, such as transplantation, macrophage subsets appear to transdifferentiate into lymphatic endothelial cells for incorporation into the lymphatic vessels^15, 16^. In a mouse corneal transplant model, macrophages express lymphatic vessel markers and contribute to inflammation-dependent corneal lymphangiogenesis^15^. In renal transplantation, recipient-derived circulating macrophages may be incorporated into the lymphatic system of the transplanted organ^16^.

Previously, it was thought that pericytes were derived from the mesenchymal cells that resided in the connective tissues surrounding blood vessels or from neural crest cells^17–22^. However, little is known about the origin of cerebrovascular pericytes and the mechanism underlying their recruitment to cerebral blood vessels. Here, we show a novel source of cerebrovascular pericytes in the very early phase of CNS vascular development. We describe CD31^+^F4/80^+^ cells that primarily function as phagocytes and express several macrophage markers. These cells are observed to adhere to the newly formed subventricular vascular plexus (SVP), divide into daughter cells, and eventually transdifferentiate into NG2/PDGFRβ/desmin-expressing cerebrovascular pericytes. Therefore, in the very early phase of CNS vascular development, we conclude that a subset of cerebrovascular pericytes is recruited by blood flow from sites of embryonic hematopoiesis, such as the yolk sac, and derive from the CD31^+^F4/80^+^ cells, a subset of mature macrophages.

## Results

### A subset of mature macrophages associates with cerebral blood vessels and expresses pericyte markers

**Figure 1:**
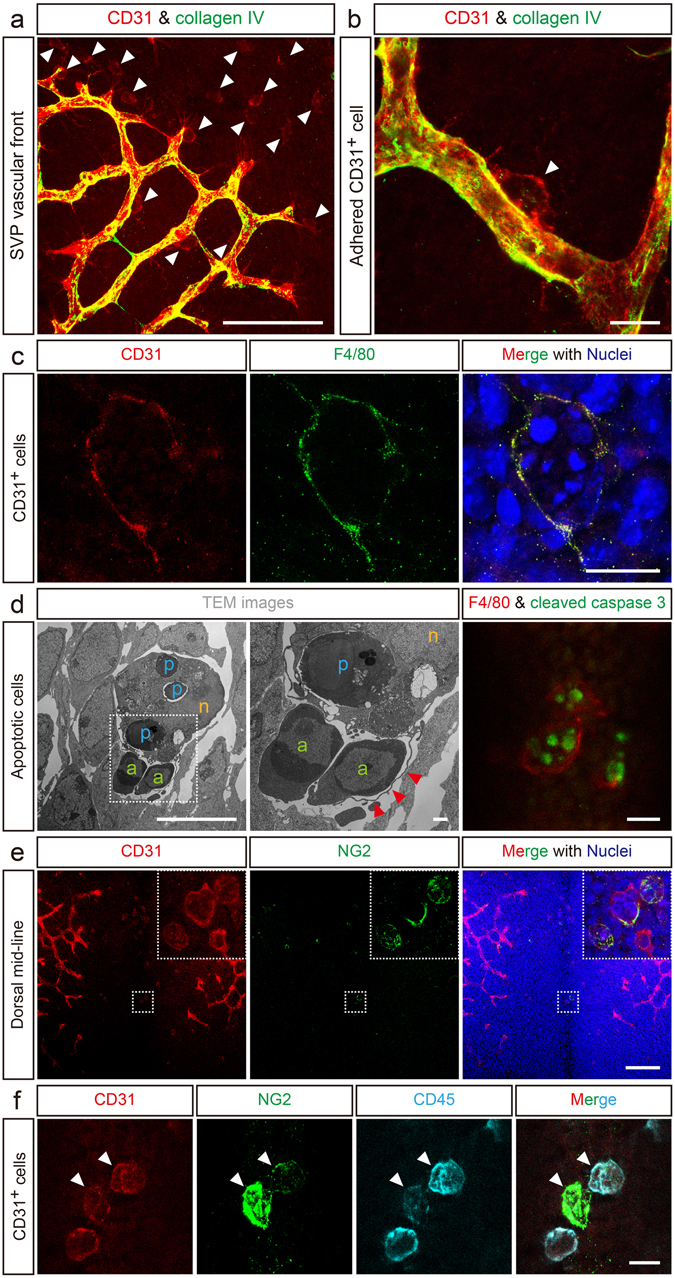
CD31^+^F4/80^+^ cells have a mature macrophage phenotype and express a pericyte marker. (**a**) CD31^+^ cells infiltrate the developing subventricular vascular plexus (SVP) vascular front (red, arrowheads). Collagen type IV marks the blood vessels (green). (**b**) High magnification image of an adhered CD31^+^ cell (red, arrowhead). (**c**) CD31^+^ cells in the dorsal midline express F4/80 (green). The merged image with stained nuclei (blue) shows the mature phagocytic macrophage morphology. (**d**) Representative image of CD31^+^F4/80^+^-equivalent phagocytes as observed by TEM. The highest magnification image is shown in the middle. Red arrowheads indicate a pseudopod, a structure typical of mature phagocytic macrophages. a, apoptotic cells (green); p, phagocytosed apoptotic cells (blue); n, nucleus (orange). Right panel in (**d**), CD31^+^F4/80^+^ cells phagocytose apoptotic cells. (**e**) CD31^+^ cells (red) express the pericyte marker NG2 (green). Insets are high magnification images of the area inside the dotted squares. (**f**) CD31^+^ cells (red) co-express CD45 (cyan) and NG2 (green). Scale bars represent 100 μm (**a** and **e**); 10 μm (**b**,** c**,** d** left and right), and (**f**); and 1 μm (**d**, middle).

During neurogenesis in mice, considerable formation of the perineural vascular plexus (PNP) and subventricular vascular plexus (SVP) occurs from embryonic day 9.5 (E9.5) to E12.5, as shown by previous study^23^ (Supplementary Figure 1a). We precisely observed the newly-formed SVP front using confocal microscopy at E10.5 (Figure 1a, Supplementary Figure 1b and c). At this time point, cells positive for CD31 and negative for collagen type IV, a well-known blood vessel-specific extracellular matrix component, surrounded the SVP front (Figure 1a, arrowheads). These CD31^+^ cells were large, had various morphologies, and were sometimes found adhered to the capillaries at the vascular front (Figure 1a, arrowheads). High magnification images showed that these CD31^+^ cells adhered to brain microcapillaries, implying that CD31^+^ cells may differentiate into cerebrovascular pericytes (Figure 1b, arrowhead).

Because the CD31^+^ cells that infiltrated the avascular area of the dorsal midline were relatively large and had varied morphologies (Figure 1a, Supplementary Figure 2a–c), we examined whether these cells expressed macrophage markers. Multi-color immunofluorescence analysis revealed that these CD31^+^ cells expressed F4/80 (Figure 1c), a marker of mature macrophages in mice^24^. The CD31^+^F4/80^+^ cells also expressed other macrophage markers such as CD206 and CD11b, suggesting that CD31^+^F4/80^+^ cells were functionally differentiated macrophages (Supplementary Figure 3). Terminally differentiated mature macrophages can engulf apoptotic cells^25, 26^. Transmission electron microscopy showed that the CD31^+^F4/80^+^ cells at the dorsal midline could phagocytose apoptotic cells with their pseudopodia (Figure 1d). In addition, cleaved caspase 3-positive apoptotic cells were phagocytosed by the F4/80^+^ phagocytic cells located in the dorsal midline area which were corresponding to the CD31^+^F4/80^+^ cells based on the distinct tissue localization and unique phagocyte-like morphology in the dorsal midline of the midbrain (Figure 1d, right).

Several lines of experimental evidence have demonstrated the possibility of macrophage transdifferentiation^15, 16^. We next examined whether the CD31^+^F4/80^+^ cells expressed a pericyte marker. Whole mount immunostaining of the brain at E10.5 showed that the CD31^+^ cells located at the dorsal midline, corresponding to the CD31^+^F4/80^+^ cells, expressed both NG2 (Figure 1e), a well-accepted early pericyte marker^1, 27^, and the pan-leukocyte marker CD45 (Figure 1f, arrowheads). These results suggest that CD31^+^F4/80^+^ cells may transdifferentiate from hematopoietic cell-derived mature macrophages into cerebrovascular pericytes.

### CD31^+^F4/80^+^ cells transdifferentiate into cerebrovascular pericytes

**Figure 2:**
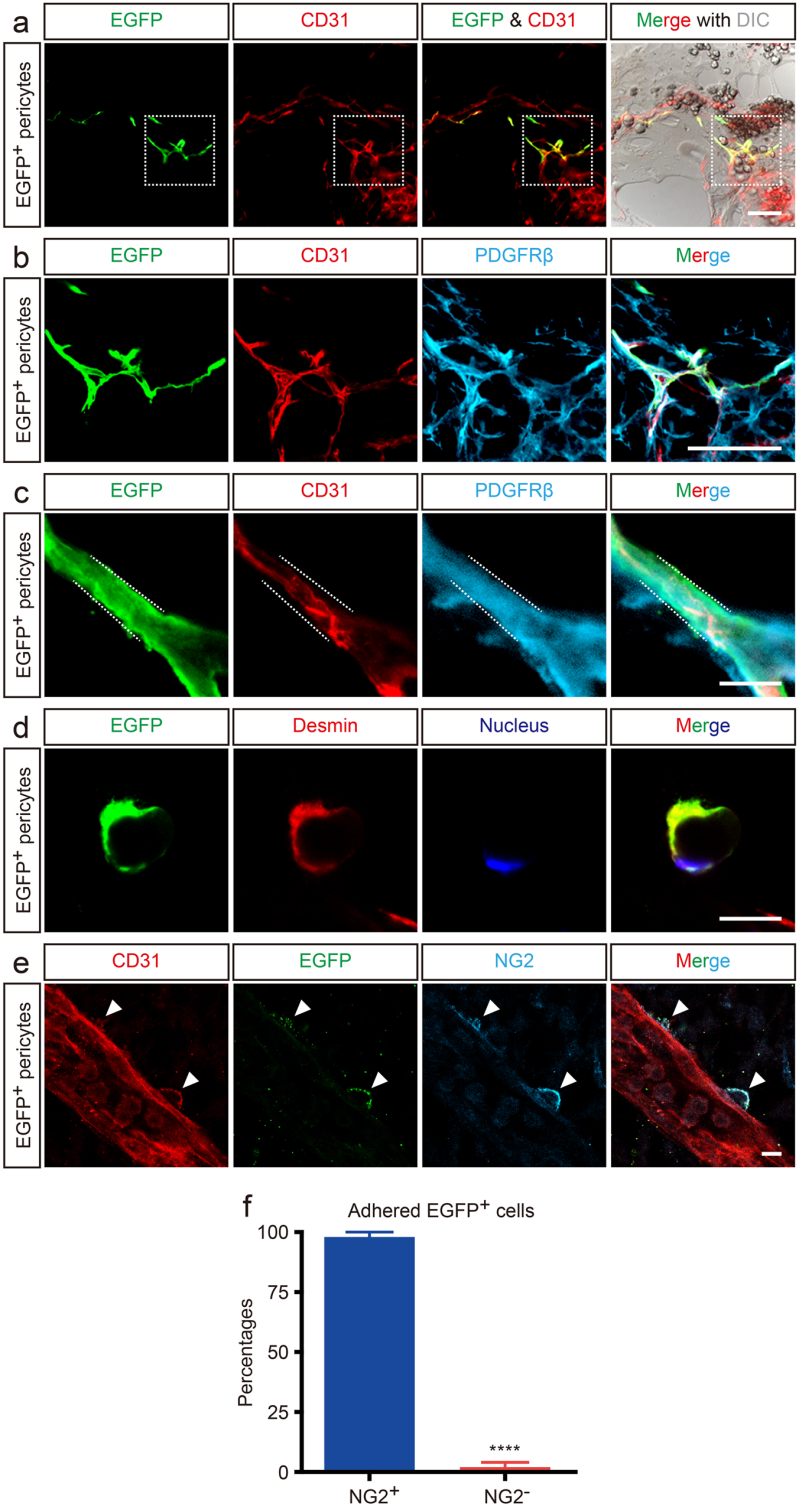
Matrigel plug and explant culture assays on CD31^+^F4/80^+^ cells. (**a**) EGFP^+^CD31^+^F4/80^+^ cells were embedded into Matrigel followed by subcutaneous injection into mice. EGFP^+^ cells associate with newly formed blood vessels. Merge with DIC image shows the appearance of the Matrigel plug. (**b**) High magnification images of the boxed area in (**a**). EGFP^+^ cells express the pericyte marker PDGFRβ (cyan). (**c**) EGFP^+^ cells (green) express PDGFRβ (cyan), instead of CD31 (red) in the higher magnification image. (**d**) Cross-section of a capillary. The transplanted EGFP^+^ cell (green) enwraps the capillary and expresses the pericyte marker desmin (red). Nucleus are counterstained with Hoechst (blue). (**e**) Explant culture assay of EGFP^+^CD31^+^F4/80^+^ cells. EGFP^+^CD31^+^F4/80^+^ cells were injected into age-matched WT embryos. EGFP^+^ cells adhere to a host cerebral blood vessel (arrowheads) and express NG2 (cyan, arrowheads). (**f**) EGFP^+^ cells that adhere to cerebral blood vessels express NG2 (****P < 0.0001; n = 3). Scale bars represent 100 μm (**a** and **b**), and 10 μm (**c**,** d** and **e**). All error bars indicate the mean ± s.e.m.

To determine whether CD31^+^F4/80^+^ cells transdifferentiate into pericytes in an *in vivo* model, we performed a Matrigel plug assay. CD31^+^F4/80^+^ cells that were fractionated from *Egfp Tg* ^28^ embryos by flow cytometry (Supplementary Figure 4) were subcutaneously administered in Matrigel to a wild type (WT) mouse. Two weeks later, the immunostained Matrigel sections showed that EGFP^+^ cells, which were the fractionated CD31^+^F4/80^+^ cells from the *Egfp Tg* mice, were tightly associated with the newly-formed host-derived capillaries (Figure 2a), and these cells also expressed the pericyte markers PDGFRβ (Figure 2b and c) and desmin (Figure 2d). The mature EGFP^+^ pericytes might not express CD31 or below the detection limit as determined by immunostaining.

We utilized another set of an experiment using an explant culture model to clarify whether CD31^+^F4/80^+^ cells transdifferentiate into pericytes. Fractionated EGFP^+^CD31^+^F4/80^+^ cells were injected into the cerebral ventricles of E10.5 WT mouse embryos. Two days later, immunostained brain specimens showed that the EGFP^+^ cells had adhered to the cerebral blood vessels and expressed the pericyte marker NG2 (Figure 2e). Statistical analysis indicated that more than 95% of the EGFP^+^ cells that adhered to the cerebral blood vessels transdifferentiated into NG2^+^ pericytes (Figure 2f). These data strongly suggest that CD31^+^F4/80^+^ cells possess the ability to transdifferentiate into pericytes in various extracellular milieus both *in vivo* and *ex vivo*.

**Figure 3:**
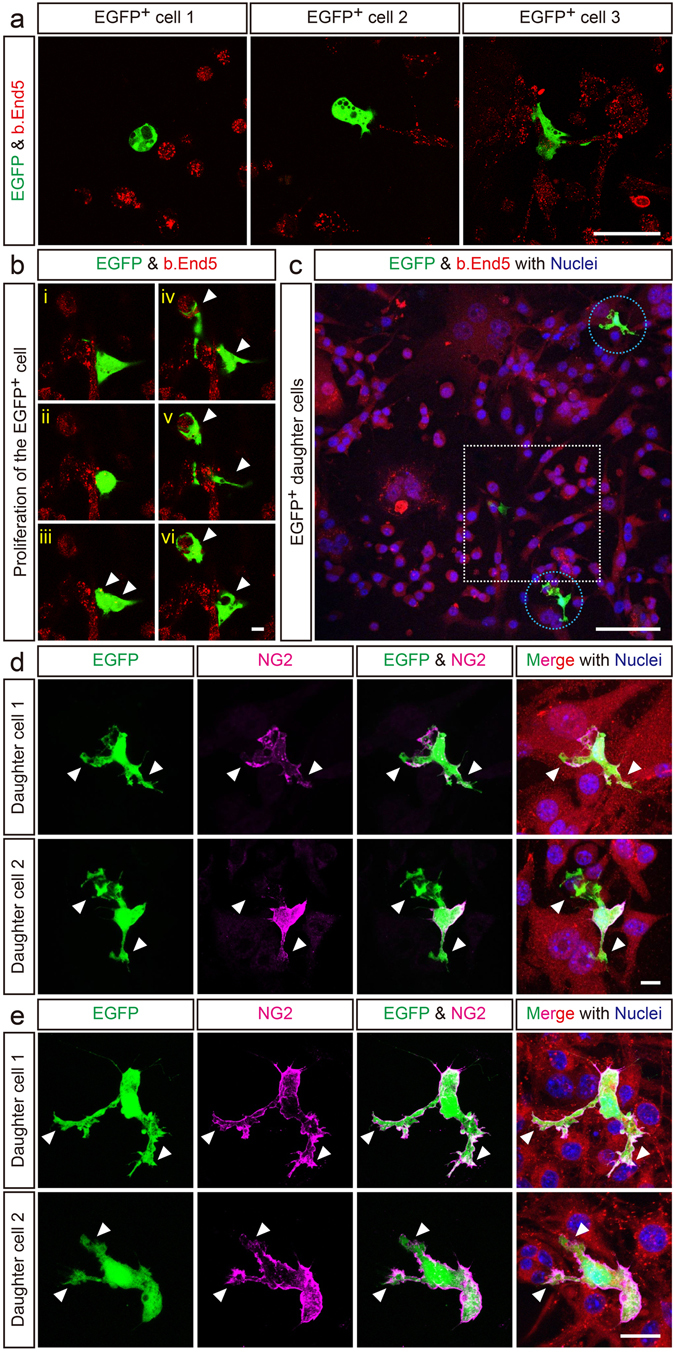
Single-cell-tracing time-lapse analysis of CD31^+^F4/80^+^ cells. (**a**) The single-cell-tracing time-lapse clearly shows that the EGFP reporter cells (equivalent to the CD31^+^F4/80^+^ cells) undergo transdifferentiation into pericytes. EGFP reporter cells (green) were cultured with b.End5 cells (red). Initially, the EGFP reporter cells exhibited various morphologies when they were seeded onto b.End5 cells. Black spots indicate phagocytosed apoptotic cells. (**b**) During the single-cell-tracing time-lapse, the EGFP reporter cells proliferated into daughter cells (arrowheads). (**c**) Low magnification image of the single-cell-tracing time-lapse specimen, EGFP^+^ daughter cells (circles), and a movie frame (square). (**d**) EGFP^+^ daughter cells express the pericyte marker NG2 (magenta). (**e**) EGFP^+^ daughter cells still expressed NG2 (magenta) and retained typical pericyte morphology over 3 days. VE-cadherin staining indicates b.End5 cells (red). Nuclei are counterstained with Hoechst (blue). Arrowheads indicate foot processes. Scale bars represent 50 μm (**a**), 10 μm (**b** and **d**), 100 μm (**c**), and 20 μm (**e**).

To obtain direct evidence of CD31^+^F4/80^+^ cells transdifferentiating into pericytes, we performed single-cell-tracing time-lapse analysis. This technique can provide direct evidence for transdifferentiation without losing any windows of time. CD31^+^F4/80^+^-equivalent EGFP reporter cells were isolated from the dorsal midline of the midbrain of E10.5 *Egfp Tg* mouse embryos, based on their distinct tissue localization and unique phagocyte morphology (Supplementary Figure 2). These cells were co-cultured with b.End5 cells, a mouse cerebrovascular endothelial cell-derived cell line (Figure 3a). The seeded EGFP reporter cells were able to adhere tightly to the b.End5 cells by their foot processes. During the single-cell-tracing time-lapse study, the EGFP reporter cell digested phagocytosed apoptotic cells (Supplementary Movie 1). Theoretically, terminally differentiated, mature, phagocytic macrophages do not actively proliferate under physiological conditions unless highly genetically engineered^29, 30^. However, the EGFP reporter cell divided into 2 daughter cells (Figure 3b, Supplementary Movie 1). Immediately after the time-lapse study, the EGFP^+^ daughter cells were subjected to immunostaining with an anti-NG2 antibody, which clearly showed that the EGFP^+^ daughter cells expressed NG2 and thus confirmed that the EGFP reporter cells isolated from the dorsal midline area could transdifferentiate into cerebrovascular pericytes after digesting apoptotic cells and dividing (Figure 3c and d). In addition, the EGFP^+^ daughter cells still expressed NG2 and retained typical pericyte morphology 3 days later (Figure 3e).

**Figure 4:**
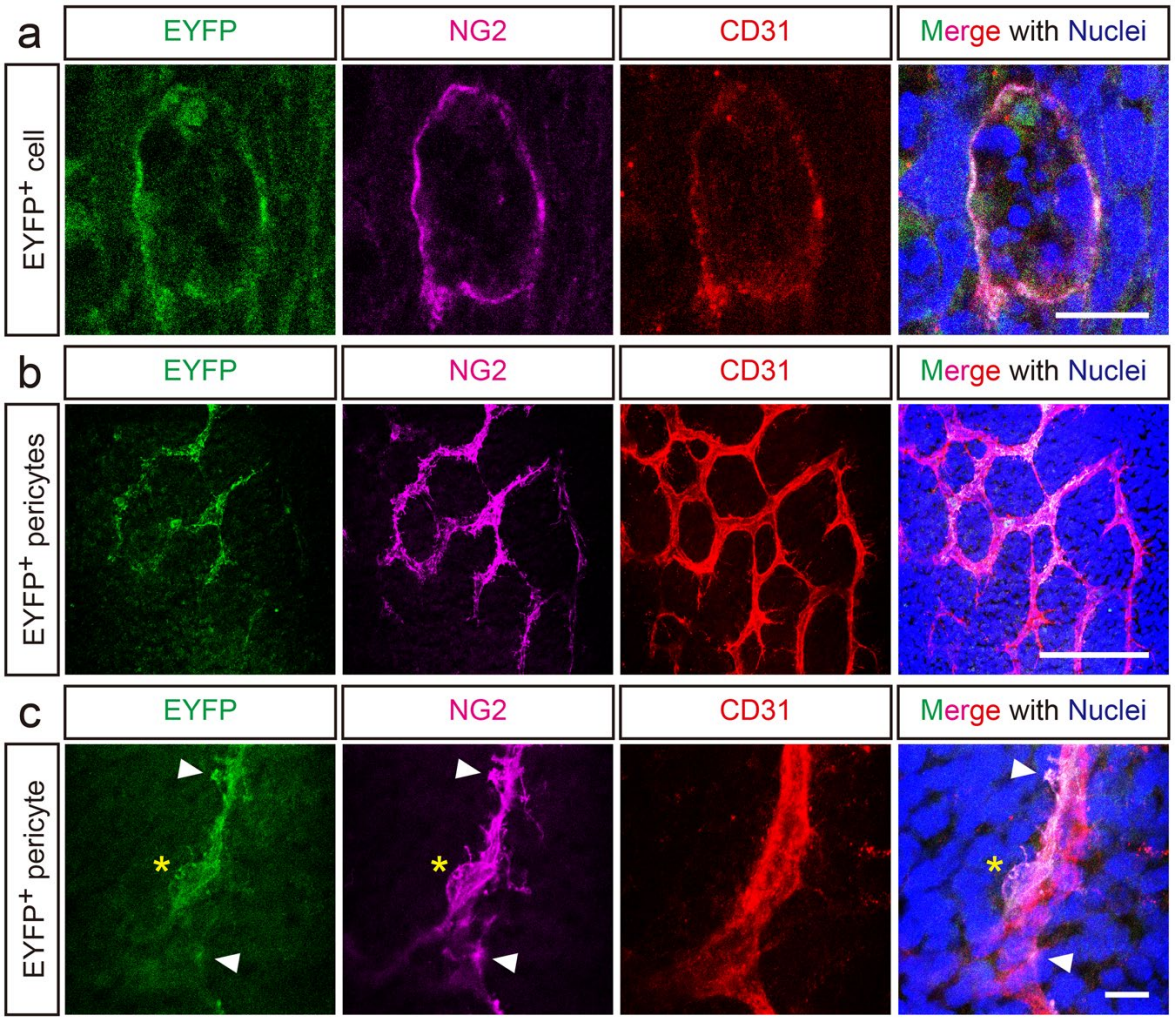
Fate mapping study of CD31^+^F4/80^+^ cells. A fate mapping study showed that CD31^+^F4/80^+^ cells originate from hematopoietic cells. (**a**) In E10.5 *R26R-Eyfp;Vav1-Cre* mouse embryos, CD31^+^ cells (red, equivalent to the CD31^+^F4/80^+^ cells), which infiltrate the dorsal midline and phagocytose apoptotic cells confirmed by multiple-nuclei (blue), express EYFP (green) and NG2 (magenta). (**b**) On the lateral side of the SVP (red), many NG2^+^ pericytes (magenta) express EYFP (green). (**c**) High magnification image of a typical pericyte expressing EYFP and NG2 (soma, asterisk; foot processes, arrowheads). Scale bars represent 10 μm (**a** and **c**) and 100 μm (**b**).

To examine whether CD31^+^F4/80^+^ cells originate from hematopoietic cells, a fate-mapping study was performed, which is a valid procedure for cell fate determination. We utilized *R26R-Eyfp;Vav1-Cre* mice, generated by crossing *R26R-Eyfp* and *Vav1-Cre* mice, which display hematopoietic cell-specific EYFP expression^31, 32^. In *R26R-Eyfp;Vav1-Cre* embryos, EYFP^+^ hematopoietic lineage cells were observed in the dorsal midline area at E10.5 and were found to co-express CD31 and NG2 (Figure 4a), indicating that these cells corresponded to the CD31^+^F4/80^+^ cells described earlier, based on their distinct tissue localization, marker expression, and unique phagocyte-like morphology. Moreover, many NG2^+^ cerebrovascular pericytes found elsewhere in the brain of *R26R-Eyfp;Vav1-Cre* embryos also expressed EYFP (Figure 4b, higher magnification in 4c).

### Cerebrovascular pericyte coverage is delayed in *Csf1*^*op/op*^ mouse

**Figure 5:**
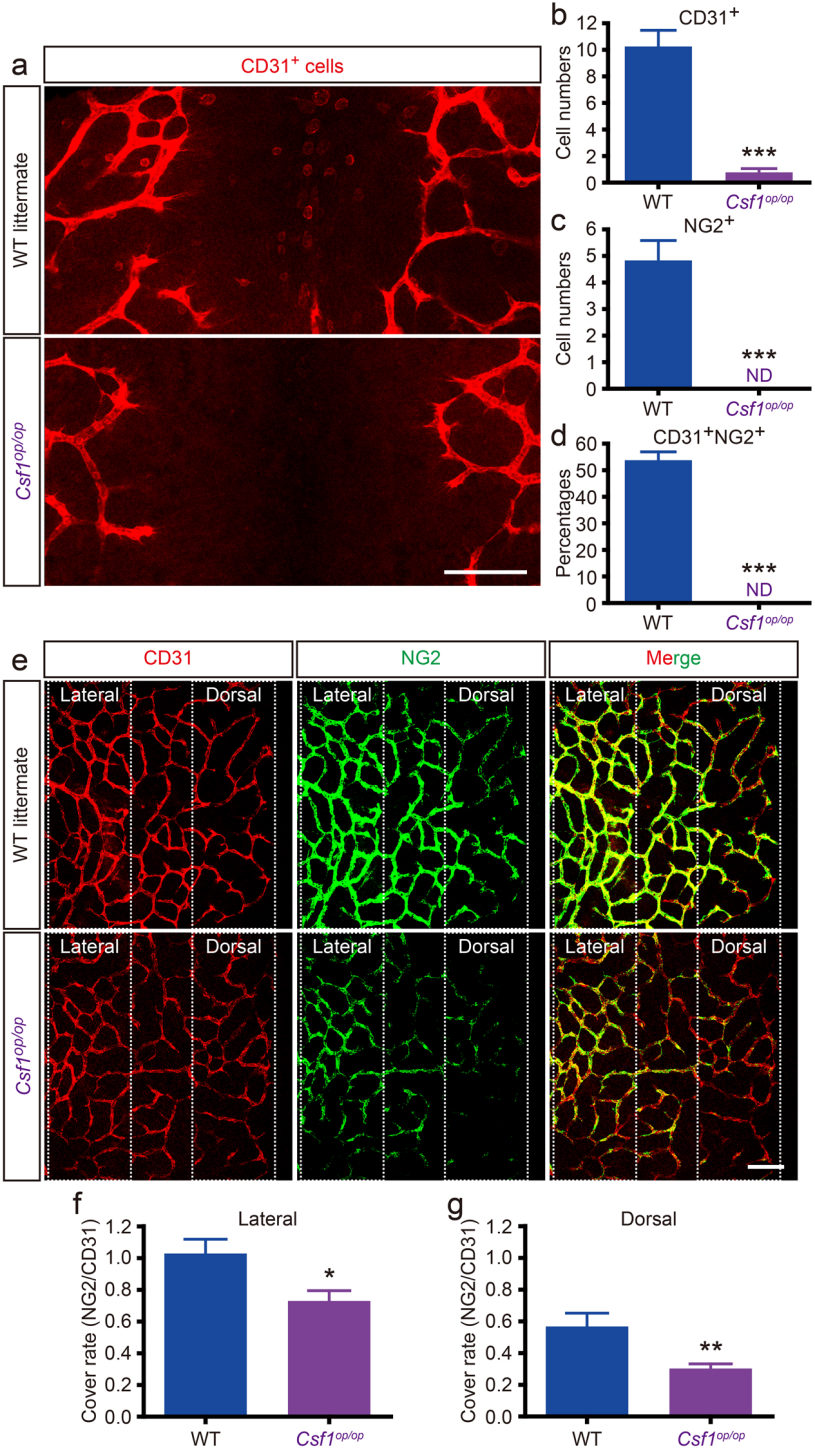
*Csf1* ^*op/op*^ mice have very few CD31^+^F4/80^+^NG2^+^cells and sparse pericyte coverage. (**a**–**d**) Very few CD31^+^NG2^+^ cells are found in the dorsal midline area of E10.5 *Csf1* ^*op/op*^ mouse embryos. While CD31^+^ cells (equivalent to the CD31^+^F4/80^+^ cells) infiltrate the dorsal midline of WT littermates, very few are observed in *Csf1* ^*op/op*^ embryos (**a**). CD31^+^ (**b**), NG2^+^ (**c**), and CD31^+^NG2^+^ (**d**) cells in both genotypes (***P < 0.001; WT = 4; *Csf1* ^*op/op*^ = 3; 200 × 200 μm, see also Supplementary Figure 5a). (**e**–**g**) Loss of NG2^+^ cells causes sparse pericyte coverage of the SVP in the *Csf1* ^*op/op*^ mouse. Although intact NG2^+^ pericyte coverage is observed in WT littermates, sparse pericyte coverage is seen in *Csf1* ^*op/op*^ mice (**e**). NG2^+^ pericyte coverage of the lateral side area (**f**) and dorsal side area (**g**) is significantly decreased in *Csf1* ^*op/op*^ mice (*P < 0.05; **P < 0.01; WT = 8; *Csf1* ^*op/op*^ = 7; 200 × 200 μm). Images for coverage analysis were rendered to the same depth (10 µm). Coverage rate was calculated as NG2 pixels/ CD31 pixels. Scale bars represent 100 μm (**a** and **b**). All error bars indicate the mean ± s.e.m.

To investigate whether CD31^+^F4/80^+^ cells contribute to the pericyte coverage of the SVP, we utilized a *Csf1* ^*op/op*^ mouse^33, 34^, which is deficient in macrophages. Compared to WT littermates, significantly fewer CD31^+^ cells were recruited to the dorsal midline in E10.5 *Csf1* ^*op/op*^ embryos (Figure 5a and b). Moreover, in *Csf1* ^*op/op*^ embryos, no NG2^+^ cells or CD31^+^NG2^+^ cells (equivalent to the CD31^+^F4/80^+^ cells) were found at the dorsal midline (Figure 5c and d, and Supplementary Figure 5a). We next observed the NG2^+^ pericyte coverage of the SVP at E10.5. Compared to WT littermates, *Csf1* ^*op/op*^ embryos showed significantly decreased pericyte coverage on the lateral side of the SVP (Figure 5e and f). Moreover, on the dorsal side of the vascular front, there was also a significantly greater difference between *Csf1* ^*op/op*^ mice and their WT littermates (Figure 5e and g). There were no significant differences in the vascular density or branching of the SVP at E10.5 between the two genotypes (Supplementary Figure 5b–d). In contrast to E10.5, on E12.5, no clear difference in pericyte coverage was observed between *Csf1* ^*op/op*^ mice and their WT littermates (Supplementary Figure 6). These data suggest that the CD31^+^F4/80^+^ cells that infiltrate the dorsal midline by E10.5 contribute to pericyte coverage of the SVP during neurovascular development. In addition, there are at least 2 pericyte sources and/or recruitment routes at this stage, enabling compensation for the lack of CD31^+^F4/80^+^ cells in the *Csf1* ^*op/op*^ mouse.

**Figure 6:**
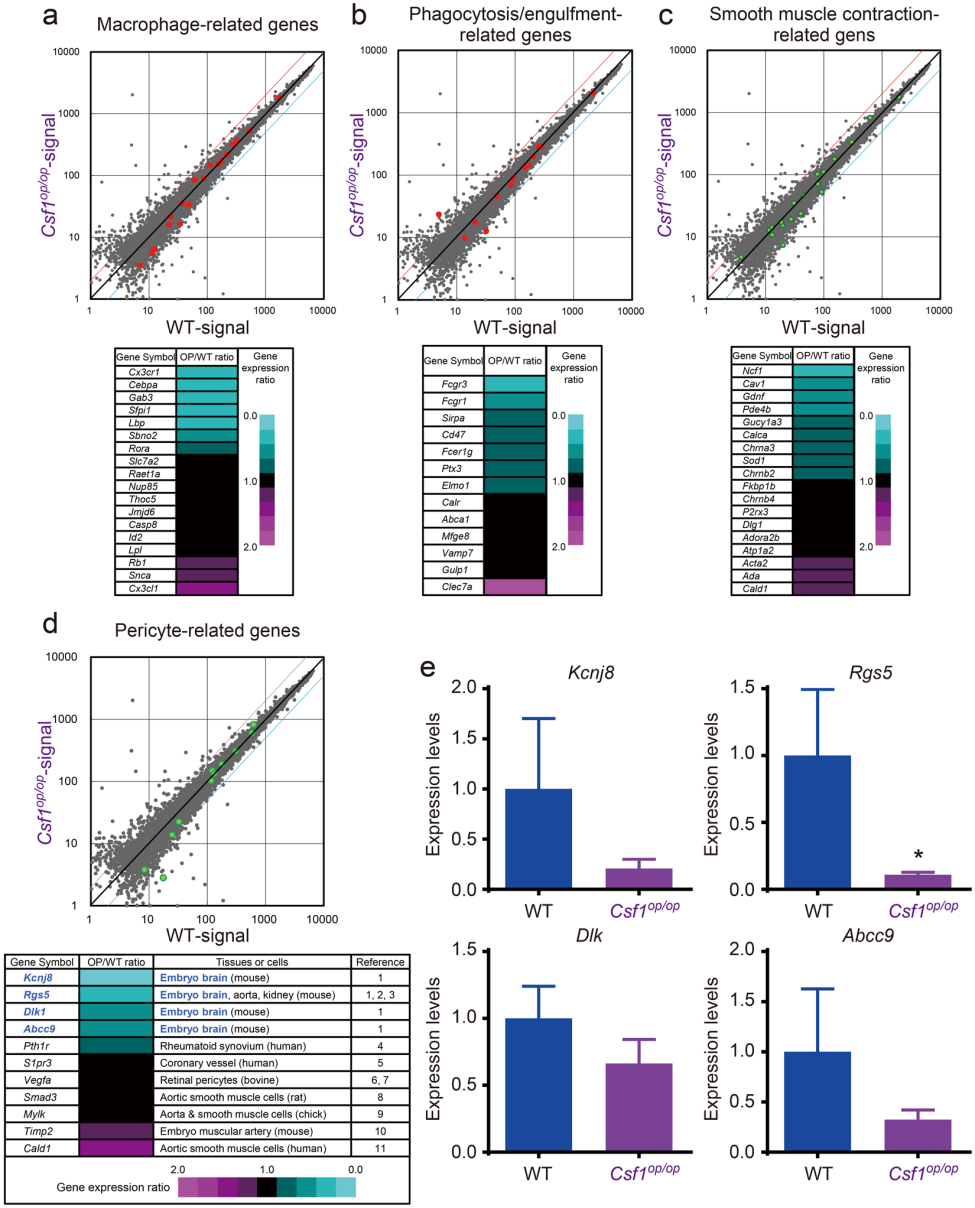
Macrophage deficiency results in down-regulation of pericyte-related genes in *Csf1* ^*op/op*^ mouse embryos. (**a**) Microarray data revealed 7 of 18 (39%) macrophage-related genes were down-regulated in the dorsal midline area of the *Csf1* ^*op/op*^ mouse embryo when compared with WT littermates. (**b**) Down-regulated phagocytosis/engulfment-related genes (7 of 13 genes, 54%), (**c**) smooth muscle contraction-related genes (9 of 18 genes, 50%), and (**d**) selected pericyte-related genes (5 of 11 genes, 45%) in the dorsal midline area of the *Csf1* ^*op/op*^ mouse embryo, as compared with a WT littermate. The top 4 down-regulated genes in (**d**), *Kcnj8, Rgs5, Dlk1*, and *Abcc9*, have been reported as cerebrovascular pericyte markers in mouse embryo. (**e**) Expression of *Kcnj8*, *Rgs5*, *Dlk1*, and *Abcc9* were confirmed by real-time PCR (*P < 0.05; WT = 3; *Csf1* ^*op/op*^ = 4). All error bars indicate the mean ± s.e.m.

To confirm whether the expression levels of pericyte-related genes are reduced in *Csf1* ^*op/op*^ mice, we performed a microarray analysis using mRNA extracted from the dorsal midline area of an E10.5 *Csf1* ^*op/op*^ mouse and WT littermate. The expression of macrophage-related genes and phagocytosis/engulfment-related genes (Gene Ontology terms) in the dorsal midline of the *Csf1* ^*op/op*^ mouse were 39% and 54% lower, respectively, than in the WT littermate (Figure 6a and b). It has been reported that cerebral pericytes can contract to regulate capillary blood flow^4, 5^. The *Csf1* ^*op/op*^ mouse embryo showed 50% lower expression of smooth muscle contraction-related genes compared to the expression levels in the WT littermate (Figure 6c). Moreover, lower expression of selected pericyte-related genes was observed in the *Csf1* ^*op/op*^ embryo compared to the WT littermate embryo (Figure 6d). The microarray data were supported by real-time PCR experiments. Compared to WT littermates, *Csf1* ^*op/op*^ mouse embryos showed lower expression of the top 4 down-regulated pericyte-related genes, *Kcnj8, Rgs5, Dlk1*, and *Abcc9* (Figure 6e), suggesting that the pericyte deficiency may be caused by a macrophage deficiency. To confirm whether CD31^+^F4/80^+^ cells are involved in angiogenesis, we reassessed the microarray data. The expression of angiogenesis-related genes in the dorsal midline of the *Csf1* ^*op/op*^ mouse showed downregulation of some genes; however, the overall expression level of angiogenesis-related genes basically appeared similar to the WT littermate (Supplementary Figure 7). These data, in combination with the previously described observations on vascular density and branching (Supplementary Figure 5), suggest that CD31^+^F4/80^+^ cells may not primarily promote angiogenesis at E10.5.

To determine whether another major hematopoietic cell subgroup, lymphocytes, contributes to cerebrovascular pericyte development during this time, we utilized the lymphoid-lineage-deficient *Rag2* ^−/−^ mouse^35^. *Rag2* mRNA was found to be expressed in the major embryonic hematopoietic organs, such as the aorta-gonad-mesonephros (AGM) region and the yolk sac, at E10.5 (Supplementary Figure 8a). Immunofluorescence on *Rag2* ^−/−^ and WT littermate embryos revealed no difference in NG2^+^ cells (equivalent to the CD31^+^F4/80^+^ cells, Supplementary Figure 8b and c). Furthermore, statistical analysis demonstrated that there were no significant differences in the numbers of NG2^+^, F4/80^+^, NG2^+^F4/80^+^, NG2^+^CD31^+^, or NG2^+^CD31^+^F4/80^+^ cells between *Rag2* ^−/−^ and WT mice (Supplementary Figure 8c). The only significant difference observed was in the number of CD31^+^ cells. These data strongly suggest that macrophages, but not lymphoid cells, contribute to pericyte development in the SVP.

### CD31^+^F4/80^+^ cells are recruited from the yolk sac by blood flow and directly infiltrate the dorsal midline before potentially transdifferentiating into pericytes

**Figure 7:**
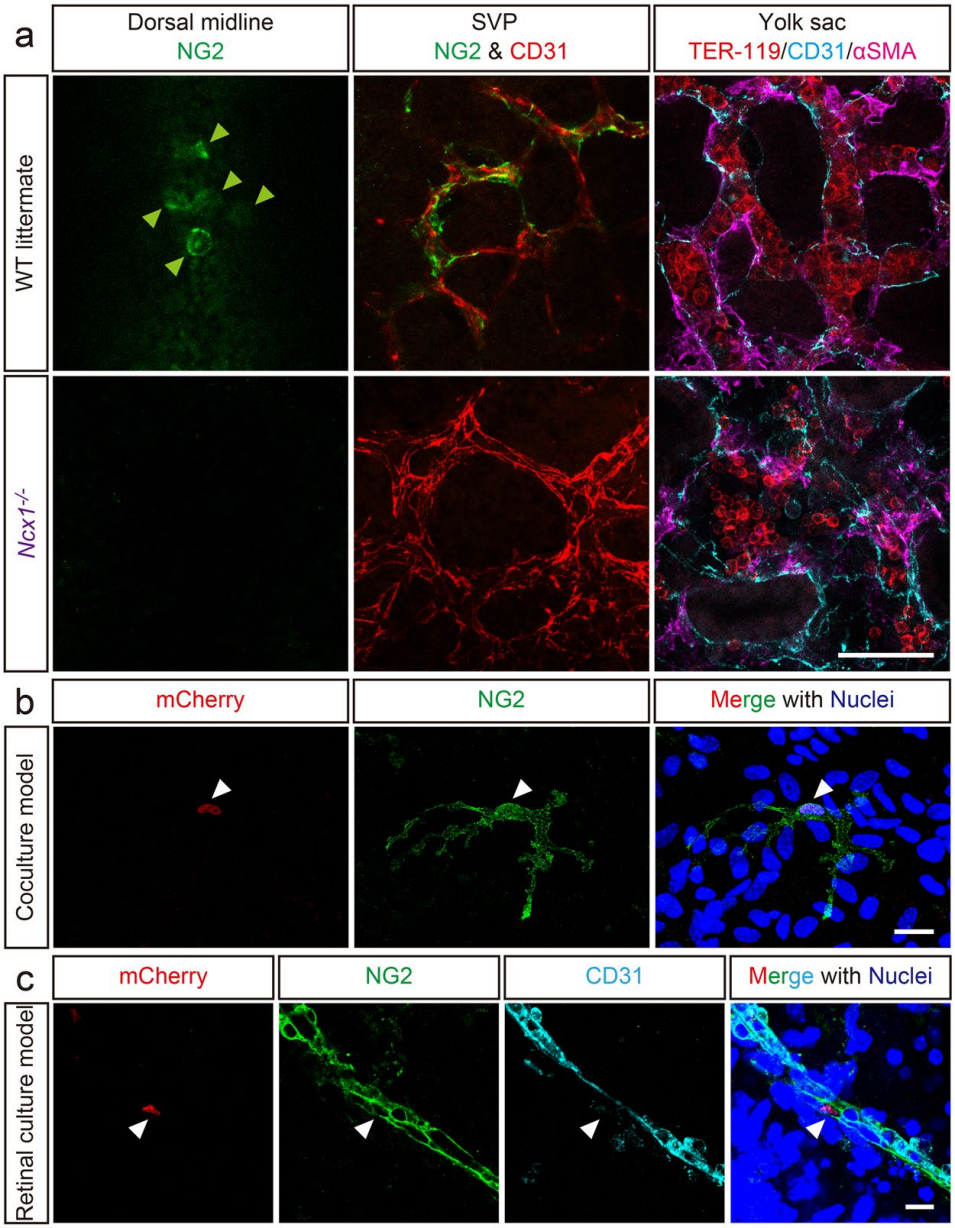
NG2^+^ cells are absent from the dorsal midline of *Ncx1* ^−/−^ mice and CD31^+^F4/80^+^NG2^−^ cells fractionated from yolk sac transdifferentiate into NG2^+^ pericytes in culture models. (**a**) Immunofluorescence of *Ncx1* ^−/−^ mice shows no recruitment of the NG2^+^ cells (equivalent to the CD31^+^F4/80^+^ cells) to the dorsal midline (green, arrowheads). While NG2^+^ pericytes (green) associated with the SVP (red) in WT mice, this was not observed in *Ncx1* ^−/−^ mice. Additionally, vascular meshworks appear to be diminished and relatively larger blood vessels can be observed. In *Ncx1* ^−/−^ yolk sacs, hematopoiesis (confirmed by TER-119^+^ embryonic erythrocytes) is not substantially diminished. (**b** and **c**) CD31^+^F4/80^+^NG2^−^ cells fractionated from yolk sacs of mCherry^+^ embryos, which constitutively express nuclear-localized mCherry protein, transdifferentiate into NG2^+^ pericytes. (**b**) mCherry^+^CD31^+^F4/80^+^NG2^−^ cells (red) are cultured with b.End5 cells and neural stem/progenitor cells. On 7DIV, the mCherry^+^ cells (red, arrowhead) express NG2 (green) and show foot processes, a typical structure of mature pericytes. (**c**) Following 3 days of retinal explant culture with mCherry^+^CD31^+^F4/80^+^NG2^−^ cells, the mCherry^+^ cells (red, arrowhead) express NG2 (green), and enwrap retinal blood vessels (cyan). Nuclei are counterstained with Hoechst (blue). Scale bars represent 50 μm (**a**), 20 μm (**b**), and 10 μm (**c**).

To clarify the hematopoietic site of origin of the CD31^+^F4/80^+^ cells, we used an *Ncx1* ^−/−^ mouse^36, 37^, which has a non-beating heart. The lack of blood flow resulted in no hematopoietic cells in the dorsal aorta or the cutaneous microvasculature, particularly TER-119^+^ embryonic erythrocytes, a major blood cell type at E10.5 (Supplementary Figure 9a and b). In *Ncx1* ^−/−^ embryos, the lack of blood flow resulted in no NG2^+^ cells (equivalent to the CD31^+^F4/80^+^ cells) recruitment to the dorsal midline area at E10.5 (Figure 7a and Supplementary Figure 9c). In addition, no NG2^+^ pericytes were observed in the SVP of *Ncx1* ^−/−^ mouse embryos (Figure 7a). However, in contrast to other sites, yolk sac hematopoiesis was not substantially diminished in the mutant embryos (Figure 7a and Supplementary Figure 9d). These data strongly suggest that CD31^+^F4/80^+^ cells are recruited from an extraembryonic hematopoietic organ, the yolk sac. By a more direct method, CD31^+^F4/80^+^NG2^−^ cells were fractionated from the yolk sacs of *R26-mCherry* embryos by flow cytometry (Supplementary Figure 9e) and co-cultured with b.End5 cells and neural stem/progenitor cells. Immunofluorescence data showed that mCherry^+^ cells, which derived from CD31^+^F4/80^+^NG2^−^ cells, expressed NG2 at 7 days *in vitro* (DIV) (Figure 7b). Moreover, these mCherry^+^ cells were found to both express NG2 and enwrap blood vessels in a retinal explant model (Figure 7c). These results suggest that hematopoietic lineage cells isolated from the yolk sac transdifferentiated into functionally mature pericytes that associated with vascular endothelial cells both *in vitro* and *ex vivo*.

**Figure 8:**
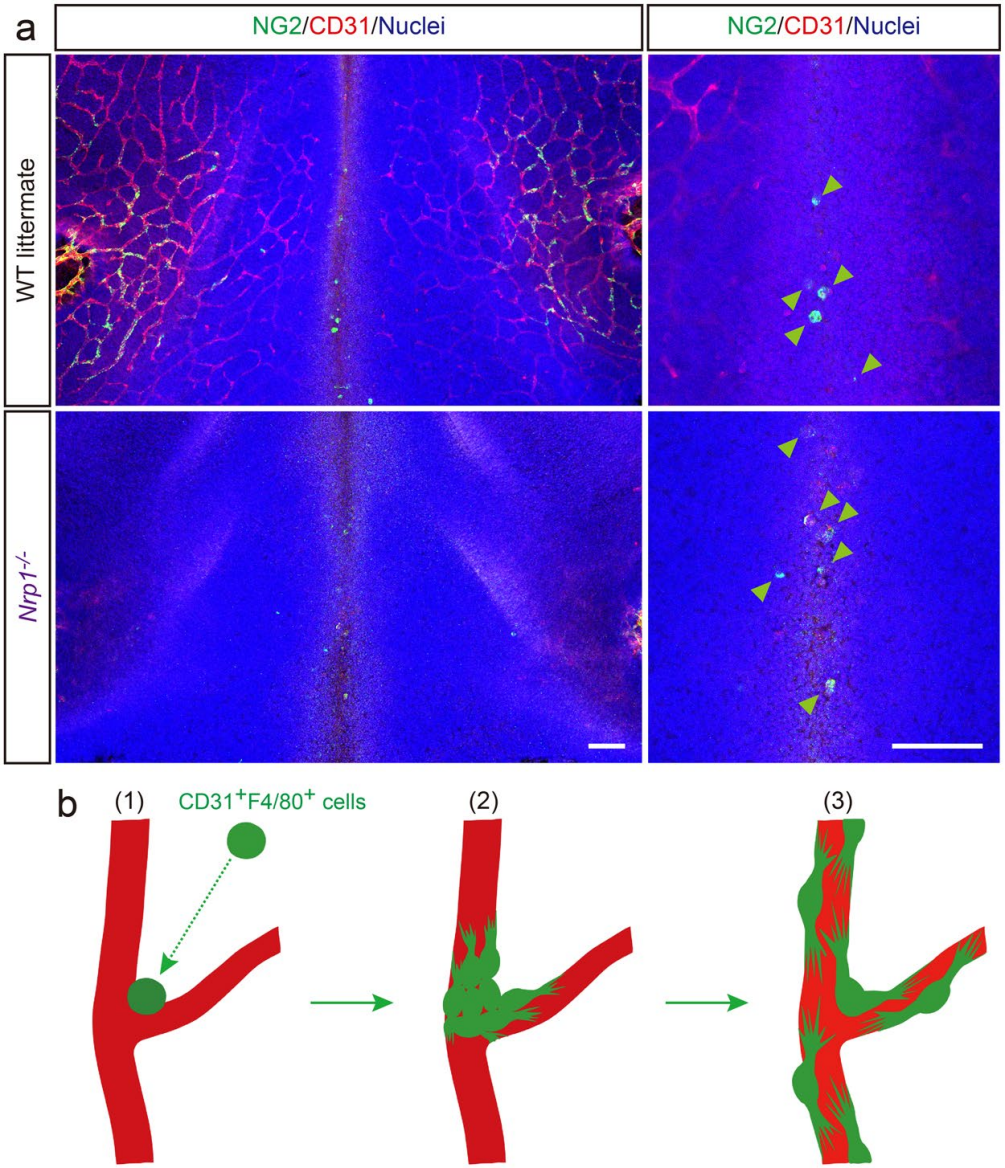
*Nrp1* ^−/−^ mice show intact recruitment of hematopoietic-lineage pericytes. (**a**) *Nrp1* ^−/−^ mice show intact recruitment of the NG2^+^ cells (green, equivalent to the CD31^+^F4/80^+^ cells) to the dorsal midline area, similar to WT littermates (WT, 4.33 ± 0.67; *Nrp1* ^−/−^, 5.18 ± 0.75). CD31 staining delineates SVP (red). Nuclei are counterstained with Hoechst (blue). Statistical analysis of NG2^+^ cell recruitment shows no significant difference between the 2 genotypes (WT = 5, *Nrp1* ^−/−^ = 4, 200 × 200 μm). Scale bars represent 100 μm. (**b**) Proposed scheme of macrophage-derived pericyte coverage in the SVP. (1) During the very early phase of vascular development in the CNS, CD31^+^F4/80^+^ cells (hematopoietic-lineage pericyte subset) are transported by the blood and infiltrate the dorsal midline area. Concomitant with infiltration in the neuroepithelium, CD31^+^F4/80^+^ cells express NG2 and adhere to the developing SVP. (2) CD31^+^F4/80^+^ cells proliferate, migrate along the capillaries, and transdifferentiate into mature pericytes. (3) Pericyte coverage of the SVP is completed.

Finally, to determine whether CD31^+^F4/80^+^ cells directly infiltrate the dorsal midline area or are recruited *via* the developing SVP, we utilized an *Nrp1* ^−/−^ mouse^38^, which is deficient in SVP morphogenesis. In *Nrp1* ^−/−^ embryos, NG2^+^ cells (equivalent to the CD31^+^F4/80^+^ cells) presented in the dorsal midline area on E10.5 (Figure 8a), suggesting that these cells might be transported through the conduit of the PNP by blood flow and then extravasate and infiltrate the neuroepithelium around the dorsal midline area. There was no significant difference in NG2^+^ cell infiltration between the *Nrp1* ^−/−^ and WT embryos (WT, 4.33 ± 0.67; *Nrp1* ^−/−^, 5.18 ± 0.75). Our data clearly demonstrate that there are at least two mechanisms of pericyte recruitment to the cerebral blood vessels. The first mechanism, direct recruitment to the dorsal midline, was demonstrated in this study (Figures 8b and Supplementary Figure 10). Another mechanism is locomotion along the microvessels, which is a well-known developmental process in the field of vascular biology (Supplementary Figure 10) ^22, 39–41^.

## Discussion

Our findings clearly demonstrate that a specific subset of CD31^+^F4/80^+^ macrophages infiltrate the dorsal midline at E10.5 and transdifferentiate into NG2/PDGFRβ/desmin-expressing cerebrovascular pericytes, which cover the SVP in the very early phase of CNS vascular development. These cells are recruited from the yolk sac, the most important extraembryonic hematopoietic organ in the early to middle embryonic stage. The CD31^+^F4/80^+^ cells, at first, phagocytosed apoptotic cells similarly to mature macrophages when they infiltrated the dorsal midline. Then, they began to proliferate. Theoretically, terminally differentiated, mature, phagocytic macrophages do not actively proliferate under physiological conditions^29^. Aziz and colleagues have reported that MafB/c-Maf-deficient mature macrophages can proliferate *in vitro* and *in vivo* without dedifferentiation and do not produce a tumor^30^. However, MafB/c-Maf-deficient mature macrophages are highly genetically engineered cells and their study was not performed under physiological conditions. Our data clearly demonstrate that the CD31^+^F4/80^+^ cells identified here are a subset of mature macrophages with phagocytic activity that retain proliferative ability under physiological conditions at least in the very early phase of vascular development in the CNS. Our findings also suggest that the function of CD31^+^F4/80^+^ cells appears to be involved in vascular maturation as cerebrovascular pericytes rather than angiogenic macrophages^42^.

Several lines of experimental evidence have shown that macrophage subsets appear to contribute or transdifferentiate into other cell types, especially lymphatic endothelial cells^15, 16^. In a mouse corneal transplant model, CD11b-positive macrophages contributed to inflammation-dependent corneal lymphangiogenesis as LYVE-1 or PROX-1-positive lymphatic vascular endothelium-like cells^15^. In addition, 30%–50% of CD11b-positive cells in peritoneal exudates express lymphatic endothelial cell markers such as LYVE-1, Podoplanin, or PROX-1 and give rise to vessel-like structures *in vitro*. In gender-mismatched renal transplantation, when the recipient rejects the transplant and has a high rate of overall lymphatic endothelial proliferation as well as massive chronic inflammation, recipient-derived circulating macrophages transdifferentiate into lymphatic endothelial cells in the transplanted tissue^16^. However, these findings were exclusively observed in highly pathological conditions. In contrast to these, for the first time, we demonstrate that a fully mature macrophage subset with phagocytic activity can transdifferentiate into cerebrovascular pericytes under physiological conditions.

Morphologically, pericytes have been identified in the embryonic rat brain, where they are located on the abluminal surface of the vasculature^43^. To date, it has been reported that pericytes may be a heterogeneous cellular group with various functions and origins^1, 21, 22, 44, 45^. In fact, multiple pericyte sources such as the mesenchyme and the neural crest have been previously reported^17, 18, 21, 34, 46, 47^. In an adult lineage-tracing study of mouse bone marrow transplantation, a bone marrow origin for mural cells during VEGF-A induced dermal angiogenesis was suggested^48^. However, to the best of our knowledge, we clearly demonstrate here, for the first time, that cerebrovascular pericytes are derived from hematopoietic organs such as the yolk sac under physiological conditions in the very early phase of vascular development in the CNS. Furthermore, hematopoietic stem/progenitor cells lining the stem cell niche of such an organ could be recruited by blood flow to the developing brain as a hematopoietic lineage pericyte subset *via* functionally mature macrophages. In the very early phase of CNS vascular development, although the contribution of this hematopoietic lineage pericyte subset in the CNS vasculature may be involved in vascular maturation, the precise function and the mutual complementation with other pericytes derived from different origins remains to be determined.

Some reports have demonstrated the importance of pericytes in microvascular homeostasis^4–8, 49^. Under physiological conditions, pericytes play a pivotal role in blood flow regulation in cerebral and retinal microcapillaries^4, 5^. In addition, pericytes play a crucial role in the regulation of the BBB in adults^6–8, 11^. Conversely, pericyte loss is considered a hallmark of several diseases such as diabetic retinopathy, age-related macular degeneration, and cancer-associated retinopathy, and is correlated to the severity of the illness^50–52^. To date, many vascular regeneration studies have been performed^53–55^. However, very few have focused on pericyte recruitment to the regenerated blood vessels for accelerating blood vessel maturation. Our findings suggest that a subset of pericytes may be derived from hematopoietic stem/progenitor cells in pathological conditions as well. In the clinical field of vascular regenerative medicine, pericytes derived from hematopoietic lineage cells may induce the maturation of newly formed blood vessels to improve ischemic regions. In addition, hematopoietic lineage pericyte subset may be valuable in the treatment of unstable, leaky retinal blood vessels in patients with diabetes mellitus as part of a re-stabilization therapy^56^.

In rodent models of tumor transplantation, pericytes also play an important role in tumor development^57^. Recently, we reported that NG2-, PDGFRβ-, or αSMA-positive pericytes may be associated with tumor growth in mouse allograft and xenograft models^57^. However, although many NG2-, PDGFRβ-, or αSMA-positive pericytes are recruited to the tumor clump in WT mice, the number of pericytes and the tumor volume are significantly reduced in Flt-1 TK^−/−^ mice, which display an impairment of macrophage function^58^. Others and we have suggested that tumor pericytes contribute to tumor development and that pericyte-targeted therapy may inhibit tumor growth^56, 57, 59^. In this study, our findings suggest that anti-macrophage reagents could be used as an add-on therapeutic approach for patients with cancer at the preclinical and clinical levels, in addition to pericyte-targeted therapy.

Until now, the precise mechanism underlying the recruitment of pericytes to the cerebral microvasculature was not fully understood. Our study clearly demonstrates that there are at least 2 pericyte-recruiting mechanisms during cerebral blood vessel development (Supplementary Figure 10). One is “direct recruitment to the dorsal midline”, which was shown in this study. Another is “locomotion along the microvessels”, which is a well-accepted process in developmental biology^22, 34, 39–41^. We have tried to define the trigger for direct recruitment to the dorsal midline; however, our immunostaining and microarray-based studies exploring potential chemoattractant factors or integral receptors have not yet identified any candidates.

In conclusion, we have clearly demonstrated that the mature macrophages infiltrating the dorsal midline of the midbrain transdifferentiate into cerebrovascular pericytes in the very early phase of vascular development in the CNS. These results have defined the first wave of pericyte recruitment by identifying the cellular origin, thereby demonstrating that macrophage derived-pericytes are, at least in part, an actual component among the total cerebrovascular pericyte population at this stage. Although the contribution of this subset of pericytes in later developmental and postnatal stages remains to be determined in future study, this study opens a new avenue to the understanding of the origin and role of cerebrovascular pericytes. Our findings also suggest the possibility of macrophage-based mature/stable blood vessel regeneration therapy in the field of vascular regenerative medicine. In addition, these cells may be a new target for anti-angiogenic therapy in patients with cancer.

## Methods

### Animals

Descriptive analyses were performed on the following transgenic and knockout mouse lines: *Csf1* ^*op/op*^ ^33, 34^, *Rag2* ^−/−^^35^, *Ncx1* ^−/−^^37^, *Egfp transgenic* (*Egfp Tg*) ^28^, *Rosa26* ^*R26-mCherry*/+^ (*R26-mCherry*)^60^, *Rosa26* ^*R26R-Eyfp*/+^ (*R26R-Eyfp*);*Vav1-Cre* ^31, 32^, and *Neuropilin1* ^−/−^ (*Nrp1* ^−/−^)^38^. All experimental animal procedures were approved by the Committee for Institutional Animal Care and Use at the University of Toyama (Toyama, Japan), and experiments were carried out in accordance with the approved guidelines.

### Immunofluorescence

Immunofluorescence was performed on whole tissues, frozen sections, and cultured cells as previously described^61, 62^. Briefly, whole mount specimens were fixed in 4% paraformaldehyde on ice for 1 hour. Tissue sections were fixed in 4% paraformaldehyde, immersed in 30% PBS buffered sucrose, and embedded in OCT compound (Sakura Finetek Japan, Tokyo, Japan). Frozen sections (30 μm) were cut on a CM3000 cryostat (Leica Microsystems, Wetzlar, Germany). Cells were fixed in 4% paraformaldehyde on ice for 15 min. All specimens were permeabilized in a 0.3% Triton X-100/PBS solution and then incubated with primary antibodies diluted in 0.03% Triton X-100/PBS with 10% normal goat serum (VECTOR LABORATORIES, Burlingame, CA) overnight at 4 °C. The following primary antibodies were used for immunostaining: hamster anti-CD31 (1:200; Merck Millipore, Billerica, MA), rat anti-CD31 (1:100; BD Biosciences, San Jose, CA), rabbit anti-collagen type IV (1:500; Merck Millipore), rat anti-F4/80 (1:50; Bio-Rad Laboratories, Hercules, CA), rabbit anti-cleaved caspase 3 (1:200; Cell Signaling Technology, Danvers, MA), rabbit anti-NG2 (1:200; Merck Millipore), rat anti-CD45 (1:100; BD Biosciences), rabbit FITC-conjugated anti-GFP (1:500; Life Technologies Corporation, Carlsbad, CA), rat anti-CD105 (1:100; BD Biosciences), rat anti-CD140b (1:100; eBioscience, San Diego, CA), rabbit anti-desmin (1:500; Abcam, Cambridge, UK), rat anti-TER119 (1:100; eBioscience), mouse Cy3-conjugated anti-αSMA (1:500; Sigma-Aldrich, St. Louis, MO), rat anti-CD11b (1:20; BD Biosciences), rat Alexa647-conjugated anti-F4/80 (1:20; Bio-Rad Laboratories), and rat Alexa488-conjugated anti-CD206 (1:20; Bio-Rad Laboratories). The secondary antibodies (Ab or Fab) used were Alexa-Fluor350, Alexa-Fluor488, Alexa-Fluor568, or Alexa-Fluor633 conjugated (Life Technologies Corporation), or Cy3 or Cy5 conjugated (Jackson ImmunoResearch Laboratories, West Grove, PA), and used at dilutions of 1:250–1:500. Nuclei were stained with Hoechst 33258 (Nacalai Tesque, Koto, Japan) or TO-PRO-3 (Life Technologies Corporation). The imaging system used was a TCS SP5 confocal system (Leica Microsystems) and a BZ-9000 fluorescent microscope (KEYENCE, Osaka, Japan).

### Histology

Embryos were fixed in 4% paraformaldehyde, dehydrated, embedded in paraffin, and sectioned with a microtome. Hematoxylin and eosin staining was carried out using a standard procedure. Tissue specimens were then observed under a BX51 microscope (Olympus).

### Transmission electron microscopy

E10.5 WT embryos were fixed in a solution containing 4% formaldehyde and 2.5% glutaraldehyde in 0.1 M sodium phosphate buffer (pH 7.4) for 2 hours at room temperature and postfixed in 1% osmium tetroxide in the same buffer solution. The samples were then dehydrated in a graded series of ethanol and embedded in Epon 812 resin mixture (TAAB, Berks, UK). Ultra-thin sections approximately 70 nm thick were cut on an EM UC6 ultramicrotome (Leica Microsystems) and then stained with uranyl acetate and lead citrate. The specimens were examined with an H-7500 electron microscope (HITACHI, Tokyo, Japan).

### Cell sorting and cell culture

To prepare CD31^+^F4/80^+^ cells from E10.5 mouse brains, WT or *Egfp Tg* brains were mechanically triturated with a pipette in PBS on ice. To fractionate CD31^+^F4/80^+^NG2^−^ cells from yolk sacs, E10.5 *R26-mCherry* mouse yolk sacs were digested using Accutase (Nacalai Tesque) with 0.2% collagenase (Nacalai Tesque) and 0.005% trypsin inhibitor (Nacalai Tesque) for 1 hour at 37 °C then gently triturated with a pipette on ice. These cells were rinsed in PBS and then blocked with 3% normal mouse serum (Dako, Glostrup, Denmark) on ice for 10 min. Blocked cells were simultaneously stained with a PE-conjugated anti-F4/80 antibody (Bio-Rad Laboratories), an APC-conjugated anti-CD31 antibody (BD Biosciences) and, if needed, an Alexa488-conjugated anti-NG2 antibody (Merck Millipore) on ice for 15 min. Labeled cells were sorted using a FACSAria cell sorter (BD Biosciences). CD31^+^F4/80^+^ cells sorted from WT embryonic brains were seeded on fibronectin- or collagen type I-coated 8-well slides (BD Biosciences) and cultured in HuMedia-EG (KURABO, Osaka, Japan) for the durations indicated. CD31^+^F4/80^+^NG2^−^ cells sorted from *R26-mCherry* mouse yolk sacs were co-cultured with b.End5 cells and mouse neural stem/progenitor cells for the durations indicated.

### Matrigel plug assay

Matrigel (BD Biosciences), which was interfused with FACSAria-sorted EGFP^+^CD31^+^F4/80^+^ cells as described above, was subcutaneously injected into 8-week-old male WT C57BL/6 mice. After 2 weeks, the plugs were surgically collected, fixed, cut on a CM3000 cryostat (Leica Microsystems), and then immunostained as described above. Specimens were observed using a TCS SP5 confocal system (Leica Microsystems) and a BX51 fluorescent microscope (Olympus).

### Embryonic explant culture

FACSAria-sorted EGFP^+^CD31^+^F4/80^+^ cells, as described above, were intracerebroventricularly injected into age-matched E10.5 WT embryos. The embryos were then explant-cultured for 2 days using an EGM-2 BulletKit (Lonza, Allendale, NJ). The explant-cultured embryos were harvested, fixed, and the brains were excised for immunostaining as described above. The immunostained whole-mount specimens were observed using a TCS SP5 confocal system (Leica Microsystems) and a BX51 fluorescent microscope (Olympus).

### Retinal explant culture

FACSAria-sorted mCherry^+^CD31^+^F4/80^+^NG2^−^ cells were seeded onto flat-sheeted WT mouse retinas in an explant culture system^63^. The retinas were explant-cultured for 3 days using NeuroCult (STEMCELL Technologies, Vancouver, Canada) supplemented with 20 ng/ml FGF2 (PeproTech, Rocky Hill, NJ), 20 ng/ml EGF (PeproTech), and 2% FBS (Sigma-Aldrich). The explant-cultured retinas were fixed and immunostained as described above. The immunostained whole-mount retinas were observed using a TCS SP5 confocal system (Leica Microsystems).

### Single-cell-tracing time-lapse analysis

For single-cell-tracing time-lapse, EGFP^+^ phagocytes (see Figures 1 and 3, and Supplementary Figure 2) were manually isolated from the dorsal midline area of E10.5 *Egfp Tg* mouse embryos. PKH26-stained (Sigma-Aldrich) b.End5 cells were used as scaffold cells. Cells were maintained in a microscope stage incubator at 37 °C in a humidified atmosphere containing 5% CO_2_ throughout the experiment. Z-stack confocal images were collected in 10-minute intervals using a TCS SP5 confocal system (Leica Microsystems). Projection images and movies were obtained using LAS AF software (Leica Microsystems).

### RNA extraction, cDNA synthesis, and real-time PCR

Total RNA was isolated from E10.5 mouse dorsal midline areas using the RNeasy Mini Kit (Qiagen) according to the manufacturer’s instructions. Total RNA (1 μg) was reverse-transcribed as detailed in the PrimeScript RT reagent Kit protocol (Takara). For real-time PCR performed with an Mx3000 P (Agilent Technologies), cDNA was diluted 1:20 and PCR was performed using SYBR Premix EX Taq II (Takara). The real-time PCR program consisted of hot start enzyme activation at 95 °C for 10 sec followed by 40 cycles of amplification at 95 °C for 10 sec and 60 °C for 40 sec, as previously reported^64, 65^. Finally, to obtain the dissociation curve, a final cycle was performed at 95 °C for 1 min, 60 °C for 30 sec, and then 95 °C for 10 sec. For the data analysis, mouse *glyceraldehyde-3-phosphate dehydrogenase* (*Gapdh*) or *β-actin* (*Actb*) was used as an internal control. Induction values were calculated using MxPro analysis software (Agilent Technologies). Primer sequences are available upon request from the TAKARA BIO INC. website (http://www.takara-bio.co.jp).

### Gene expression microarray

Quantitative analysis of RNA expression was performed using Affymetrix gene chip cDNA microarrays (Affymetrix, Santa Clara, CA) according to the manufacturer’s protocol. Total RNA was prepared from E10.5 dorsal midline areas using the RNeasy Mini Kit (Qiagen, Hilden, Germany). RNA quality was measured using an Agilent 2100 Bioanalyzer (Agilent Technologies, Santa Clara, CA) to ensure the integrity of the RNA. Synthesis of cDNA, hybridization to the Affymetrix GeneChip Mouse Genome 430 2.0 Array (over 39,000 transcripts), and analysis were performed using a GeneChip 3000 Scanner (Affymetrix) according to standard protocols. The complete microarray data sets are available from the Gene Expression Omnibus (GSE46800).

### Statistical analysis

Statistical significance was determined using the Student’s *t* test. *P* values of <0.05 were considered statistically significant. Graphs were drawn using GraphPad Prism 6 (GraphPad Software, Inc., La Jolla, CA). Quantified data are presented as mean ± s.e.m. Each experiment was performed at least 3 times with similar results. Immunofluorescence studies were performed at least twice with independent staining experiments, and samples were prepared from at least 3 pregnant animals. However, only 2 individual *Ncx1* ^*−/−*^ conceptuses were utilized, prepared from 2 pregnant females.

## Electronic supplementary material

Supplementary Information

Supplementary Movie 1

## References

[1] Armulik A, Abramsson A, Betsholtz C (2005). Endothelial/pericyte interactions. Circ Res.

[2] Fujiwara T, Tenkova TI, Kondo M (1999). Wall cytoarchitecture of the rat ciliary process microvasculature revealed with scanning electron microscopy. Anat Rec.

[3] Baluk P, Hashizume H, McDonald DM (2005). Cellular abnormalities of blood vessels as targets in cancer. Curr Opin Genet Dev.

[4] Peppiatt CM, Howarth C, Mobbs P, Attwell D (2006). Bidirectional control of CNS capillary diameter by pericytes. Nature.

[5] Hall CN (2014). Capillary pericytes regulate cerebral blood flow in health and disease. Nature.

[6] Armulik A (2010). Pericytes regulate the blood-brain barrier. Nature.

[7] Daneman R, Zhou L, Kebede AA, Barres BA (2010). Pericytes are required for blood-brain barrier integrity during embryogenesis. Nature.

[8] Bell RD (2010). Pericytes control key neurovascular functions and neuronal phenotype in the adult brain and during brain aging. Neuron.

[9] Lindahl P, Johansson BR, Leveen P, Betsholtz C (1997). Pericyte loss and microaneurysm formation in PDGF-B-deficient mice. Science.

[10] Hellstrom M (2001). Lack of pericytes leads to endothelial hyperplasia and abnormal vascular morphogenesis. J Cell Biol.

[11] Shen J (2012). PDGFR-beta as a positive regulator of tissue repair in a mouse model of focal cerebral ischemia. J Cereb Blood Flow Metab.

[12] Fantin A (2010). Tissue macrophages act as cellular chaperones for vascular anastomosis downstream of VEGF-mediated endothelial tip cell induction. Blood.

[13] Stefater JA (2011). Regulation of angiogenesis by a non-canonical Wnt-Flt1 pathway in myeloid cells. Nature.

[14] Moore KJ, Tabas I (2011). Macrophages in the pathogenesis of atherosclerosis. Cell.

[15] Maruyama K (2005). Inflammation-induced lymphangiogenesis in the cornea arises from CD11b-positive macrophages. J Clin Invest.

[16] Kerjaschki D (2006). Lymphatic endothelial progenitor cells contribute to de novo lymphangiogenesis in human renal transplants. Nat Med.

[17] Rucker HK, Wynder HJ, Thomas WE (2000). Cellular mechanisms of CNS pericytes. Brain Res Bull.

[18] Hungerford JE, Little CD (1999). Developmental biology of the vascular smooth muscle cell: building a multilayered vessel wall. J Vasc Res.

[19] Heglind M (2005). Lack of the central nervous system- and neural crest-expressed forkhead gene Foxs1 affects motor function and body weight. Mol Cell Biol.

[20] Korn J, Christ B, Kurz H (2002). Neuroectodermal origin of brain pericytes and vascular smooth muscle cells. J Comp Neurol.

[21] Etchevers HC, Vincent C, Le Douarin NM, Couly GF (2001). The cephalic neural crest provides pericytes and smooth muscle cells to all blood vessels of the face and forebrain. Development.

[22] Yamanishi E, Takahashi M, Saga Y, Osumi N (2012). Penetration and differentiation of cephalic neural crest-derived cells in the developing mouse telencephalon. Dev Growth Differ.

[23] Ruhrberg C (2002). Spatially restricted patterning cues provided by heparin-binding VEGF-A control blood vessel branching morphogenesis. Genes Dev.

[24] Bertrand JY (2005). Three pathways to mature macrophages in the early mouse yolk sac. Blood.

[25] Nagata S (2010). Apoptosis and autoimmune diseases. Ann N Y Acad Sci.

[26] Geissmann F (2010). Development of monocytes, macrophages, and dendritic cells. Science.

[27] Armulik A, Genove G, Betsholtz C (2011). Pericytes: developmental, physiological, and pathological perspectives, problems, and promises. Dev Cell.

[28] Ikawa M, Yamada S, Nakanishi T, Okabe M (1999). Green fluorescent protein (GFP) as a vital marker in mammals. Curr Top Dev Biol.

[29] Klappacher GW (2002). An induced Ets repressor complex regulates growth arrest during terminal macrophage differentiation. Cell.

[30] Aziz A, Soucie E, Sarrazin S, Sieweke MH (2009). MafB/c-Maf deficiency enables self-renewal of differentiated functional macrophages. Science.

[31] de Boer J (2003). Transgenic mice with hematopoietic and lymphoid specific expression of Cre. Eur J Immunol.

[32] Srinivas S (2001). Cre reporter strains produced by targeted insertion of EYFP and ECFP into the ROSA26 locus. BMC Dev Biol.

[33] Niida S (2005). VEGF receptor 1 signaling is essential for osteoclast development and bone marrow formation in colony-stimulating factor 1-deficient mice. Proc Natl Acad Sci USA.

[34] Yoshida H (1990). The murine mutation osteopetrosis is in the coding region of the macrophage colony stimulating factor gene. Nature.

[35] Shinkai Y (1992). RAG-2-deficient mice lack mature lymphocytes owing to inability to initiate V(D)J rearrangement. Cell.

[36] Lux CT (2008). All primitive and definitive hematopoietic progenitor cells emerging before E10 in the mouse embryo are products of the yolk sac. Blood.

[37] Koushik SV (2001). Targeted inactivation of the sodium-calcium exchanger (Ncx1) results in the lack of a heartbeat and abnormal myofibrillar organization. FASEB J.

[38] Kawasaki T (1999). A requirement for neuropilin-1 in embryonic vessel formation. Development.

[39] Sato, H. *et al*. PDGFR-beta Plays a Key Role in the Ectopic Migration of Neuroblasts in Cerebral Stroke. *Stem Cells* (2015). 10.1002/stem.2212 26435273

[40] Maes C (2010). Osteoblast precursors, but not mature osteoblasts, move into developing and fractured bones along with invading blood vessels. Dev Cell.

[41] Kojima T (2010). Subventricular zone-derived neural progenitor cells migrate along a blood vessel scaffold toward the post-stroke striatum. Stem Cells.

[42] Wynn TA, Chawla A, Pollard JW (2013). Macrophage biology in development, homeostasis and disease. Nature.

[43] Fujimoto K (1995). Pericyte-endothelial gap junctions in developing rat cerebral capillaries: a fine structural study. Anat Rec.

[44] Kamouchi M, Ago T, Kuroda J, Kitazono T (2012). The possible roles of brain pericytes in brain ischemia and stroke. Cell Mol Neurobiol.

[45] Ando K (2016). Clarification of mural cell coverage of vascular endothelial cells by live imaging of zebrafish. Development.

[46] Foster K (2008). Contribution of neural crest-derived cells in the embryonic and adult thymus. J Immunol.

[47] Muller SM (2008). Neural crest origin of perivascular mesenchyme in the adult thymus. J Immunol.

[48] Rajantie I (2004). Adult bone marrow-derived cells recruited during angiogenesis comprise precursors for periendothelial vascular mural cells. Blood.

[49] Gaengel K, Genove G, Armulik A, Betsholtz C (2009). Endothelial-mural cell signaling in vascular development and angiogenesis. Arterioscler Thromb Vasc Biol.

[50] Ejaz S, Chekarova I, Ejaz A, Sohail A, Lim CW (2008). Importance of pericytes and mechanisms of pericyte loss during diabetes retinopathy. Diabetes Obes Metab.

[51] Cao R (2010). VEGFR1-mediated pericyte ablation links VEGF and PlGF to cancer-associated retinopathy. Proc Natl Acad Sci USA.

[52] Hirschi KK, D’Amore PA (1996). Pericytes in the microvasculature. Cardiovasc Res.

[53] Kumar AH, Caplice NM (2010). Clinical potential of adult vascular progenitor cells. Arterioscler Thromb Vasc Biol.

[54] Germani A, Di Campli C, Pompilio G, Biglioli P, Capogrossi MC (2009). Regenerative therapy in peripheral artery disease. Cardiovasc Ther.

[55] Nakamura T, Mizuno S (2010). The discovery of hepatocyte growth factor (HGF) and its significance for cell biology, life sciences and clinical medicine. Proc Jpn Acad Ser B Phys Biol Sci.

[56] Raza A, Franklin MJ, Dudek AZ (2010). Pericytes and vessel maturation during tumor angiogenesis and metastasis. Am J Hematol.

[57] Muramatsu M, Yamamoto S, Osawa T, Shibuya M (2010). Vascular endothelial growth factor receptor-1 signaling promotes mobilization of macrophage lineage cells from bone marrow and stimulates solid tumor growth. Cancer Res.

[58] Hiratsuka S, Minowa O, Kuno J, Noda T, Shibuya M (1998). Flt-1 lacking the tyrosine kinase domain is sufficient for normal development and angiogenesis in mice. Proc Natl Acad Sci USA.

[59] Kano MR (2007). Improvement of cancer-targeting therapy, using nanocarriers for intractable solid tumors by inhibition of TGF-beta signaling. Proc Natl Acad Sci USA.

[60] Abe T (2011). Establishment of conditional reporter mouse lines at ROSA26 locus for live cell imaging. Genesis.

[61] Yamamoto S (2015). Inflammation-induced endothelial cell-derived extracellular vesicles modulate the cellular status of pericytes. Sci Rep.

[62] Yamamoto S (2005). Essential role of Shp2-binding sites on FRS2alpha for corticogenesis and for FGF2-dependent proliferation of neural progenitor cells. Proc Natl Acad Sci USA.

[63] Moritoh S, Tanaka KF, Jouhou H, Ikenaka K, Koizumi A (2010). Organotypic tissue culture of adult rodent retina followed by particle-mediated acute gene transfer *in vitro*. PLoS One.

[64] Horikawa S (2015). PDGFRalpha plays a crucial role in connective tissue remodeling. Sci Rep.

[65] Sato H (2016). PDGFR-beta Plays a Key Role in the Ectopic Migration of Neuroblasts in Cerebral Stroke. Stem Cells.

