# Supplementary material for: A subset of cerebrovascular pericytes originates from mature macrophages in the very early phase of vascular development in CNS
Source: Sci Rep. 2017 Jun 20;7:3855. doi: 10.1038/s41598-017-03994-1 (PMC5478595; doi:10.1038/s41598-017-03994-1)
Supplement: Supplementary file 1 — Supplementary Information [file 41598_2017_3994_MOESM1_ESM.pdf]

Supplementary Information

A subset of cerebrovascular pericytes originates from mature macrophages in the very early phase of vascular development in CNS

Seiji Yamamoto[1,23,*], Masashi Muramatsu[2], Erika Azuma[1,3], Masashi Ikutani[4], Yoshinori Nagai[4,22], Hiroshi Sagara[5], Bon-Nyeo Koo[6,7], Satomi Kita[8], Erin O'Donnell[7], Tsuyoshi Osawa[9], Hiroyuki Takahashi[10], Ken-ichi Takano[11], Mitsuko Dohmoto[12], Michiya Sugimori[13], Isao Usui[14], Yasuhide Watanabe[15], Noboru Hatakeyama[16], Takahiro Iwamoto[8], Issei Komuro[17], Kiyoshi Takatsu[4,18], Kazuyuki Tobe[14], Shumpei Niida[19], Naoyuki Matsuda[20], Masabumi Shibuya[21], Masakiyo Sasahara[1]

[1]Department of Pathology, University of Toyama, Toyama, Japan

[2]Institute of Resource Development and Analysis, Kumamoto University, Kumamoto, Japan

[3]Department of Technology Development, Astellas Pharma Tech Co., Ltd., Toyama, Japan

[4]Department of Immunobiology and Pharmacological Genetics, University of Toyama, Toyama, Japan

[5]Medical Proteomics Laboratory, Institute of Medical Science, University of Tokyo, Tokyo, Japan

[6]Department of Anesthesiology, Yonsei University College of Medicine, Seoul, Korea

[7]Laboratory of Stem Cell and Neuro-Vascular Biology, Genetics and Developmental Biology Center, National Heart, Lung, and Blood Institute, National Institutes of Health, Maryland, USA

[8]Department of Pharmacology, Faculty of Medicine, Fukuoka University, Fukuoka, Japan

[9]Laboratry for Systems Biology and Medicine, Research Center for Advanced Science and Technology, The University of Tokyo, Tokyo, Japan

[10]Division for Health Service Promotion, The University of Tokyo, Tokyo, Japan

[11]Departments of Pharmacology, Weill Cornell Medical College, New York, USA

[12]Genome Biotechnology Laboratory, Kanazawa Institute of Technology, Ishikawa, Japan

[13]Department of Integrative Neuroscience, University of Toyama, Toyama, Japan

[14]First Department of Internal Medicine, University of Toyama, Toyama, Japan

[15]Faculty of Medicine, School of Nursing, Hamamatsu University School of Medicine, Shizuoka, Japan

[16]Department of Anesthesiology, Graduate School of Medicine, Aichi Medical University, Aichi,

Japan
[17]Department of Cardiovascular Medicine, The University of Tokyo Graduate School of Medicine, Tokyo, Japan
[18]Toyama Prefectural Institute for Pharmaceutical Research, Toyama, Japan
[19]Medical Genome Center, Center for Geriatrics and Gerontology, Aichi, Japan
[20]Department of Emergency and Critical Care Medicine, Nagoya University, Nagoya, Japan
[21]Department of Research and Education, Jobu University, Gunma, Japan
[22]JST, PRESTO, Kawaguchi, Saitama, Japan
[23]Ishidsu Shun Memorial Scholarship, Tokyo, Japan

*Correspondence to: Seiji Yamamoto, Ph.D., Department of Pathology, Graduate School of Medicine and Pharmaceutical Sciences, University of Toyama, Toyama 930-0194, Japan
Tel: +81-076-415-8879, Fax: +81-076-434-5016, E-mail: seiyama@med.u-toyama.ac.jp

Supplementary Figure 1

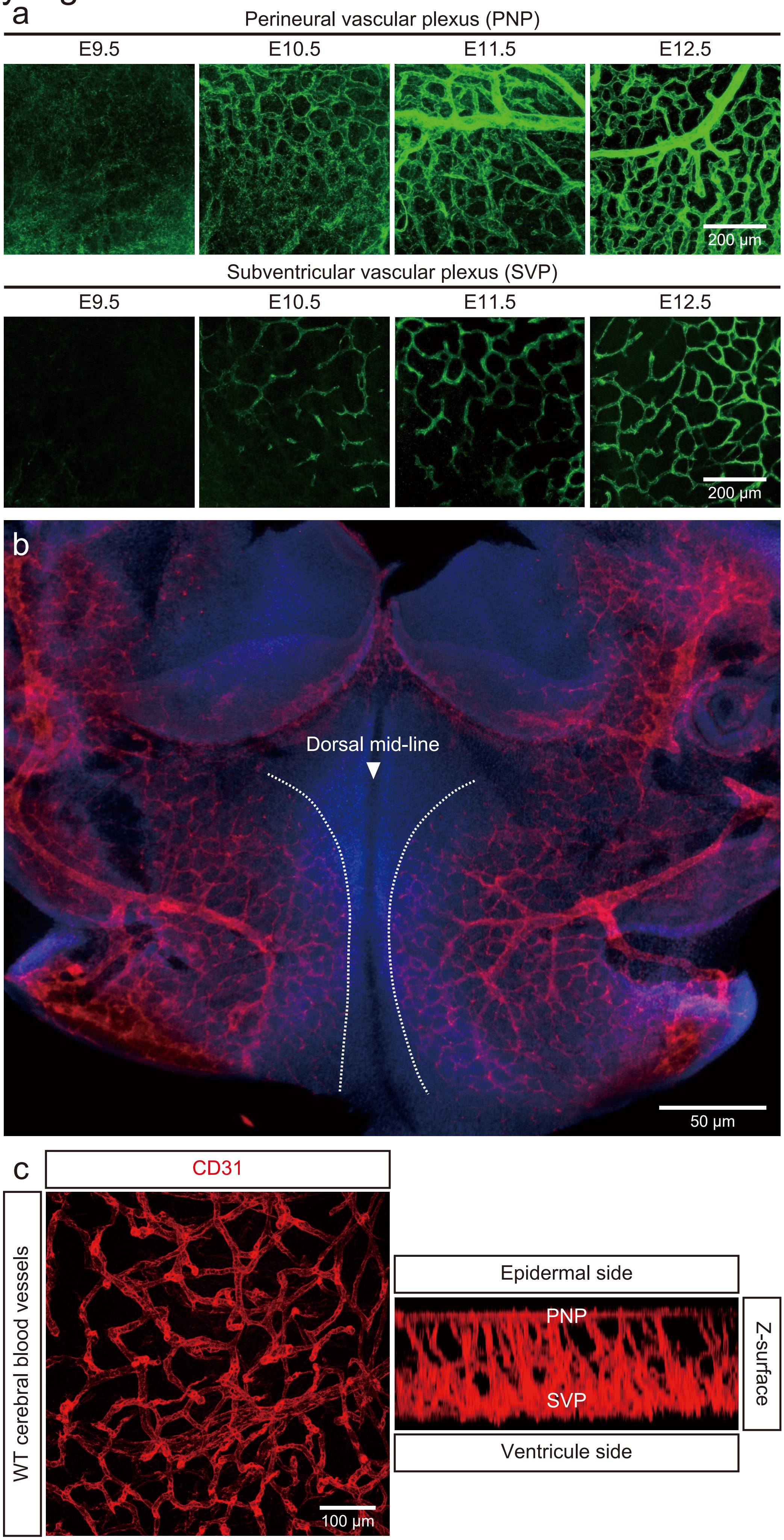

Supplementary Figure 1. PNP and SVP development from E9.5 to E12.5 in mouse embryos. (a) PNP development of the midbrain occurs from E9.5 to E12.5. Blood vessels are delineated by collagen type IV immunostaining (green). SVP development also occurs from E9.5 to E12.5 through angiogenesis. On E9.5, a few sprouts are observed in the neuroepithelium. SVP develops significantly throughout the entire avascular area of the neuroepithelium during this period. (b) Midbrain cerebral vascular formation in the E10.5 mouse embryo. CD31 staining concurrently delineates the formation of the SVP and PNP (red). Dotted lines indicate the vascular front of the SVP. In the SVP at E10.5, the dorsal midline area (arrowhead) is still avascular. Nuclei are counterstained with Hoechst (blue). (c) Rendered Z-stack confocal image showing dense vascular networks (CD31, red). Reconstituted Z-surface image depicting SVP and PNP.

Supplementary Figure 2

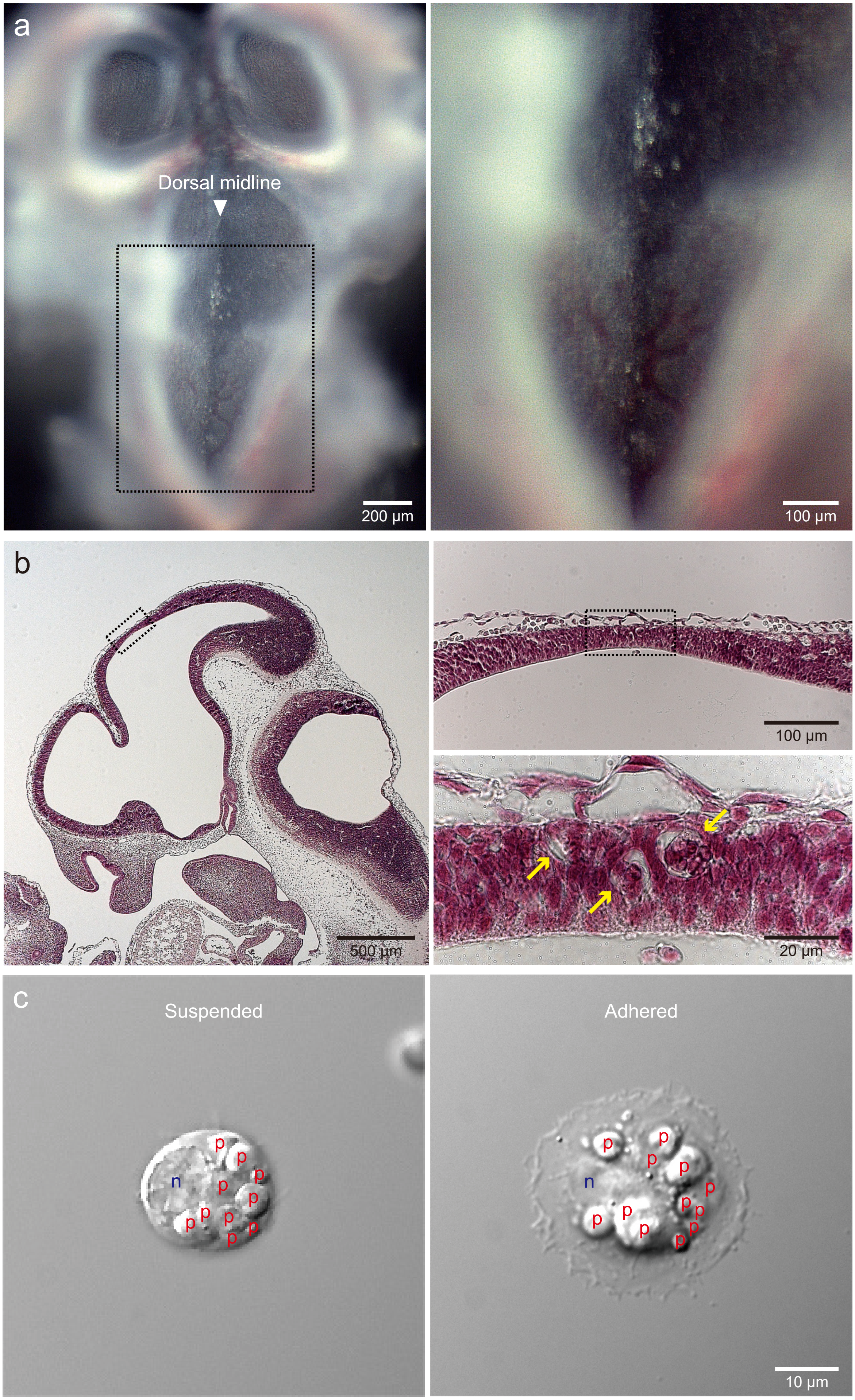

Supplementary Figure 2. The phagocytes infiltrating the dorsal midline area are equivalent to the $CD31^+F4/80^+$ cells.

(a) Ventricle side view of the E10.5 mouse brain. Relatively large, light-reflecting, complex cells are observed in the dorsal midline area. The right panel shows a high magnification image of the boxed area in the left panel. The localization, morphology, and number of phagocytes correspond to the $CD31^+F4/80^+$ cells observed in Figures 1c-f, 4a, 5a, 7a. (b) Hematoxylin and eosin stained parasagittal section of the E10.5 dorsal midline area. The boxed area is enlarged in the upper right panel and further magnified on the lower right. Arrows indicate phagocytes. (c) The phagocytes can be isolated under the microscope based on their distinct tissue localization and unique phagocyte morphology (see Figure 3a). Phagocytosed apoptotic cells are observed in the isolated phagocytes when they are either suspended (left) or adhered (right). p (red), phagocytosed apoptotic cells; n (blue), nuclei.

Supplementary Figure 3

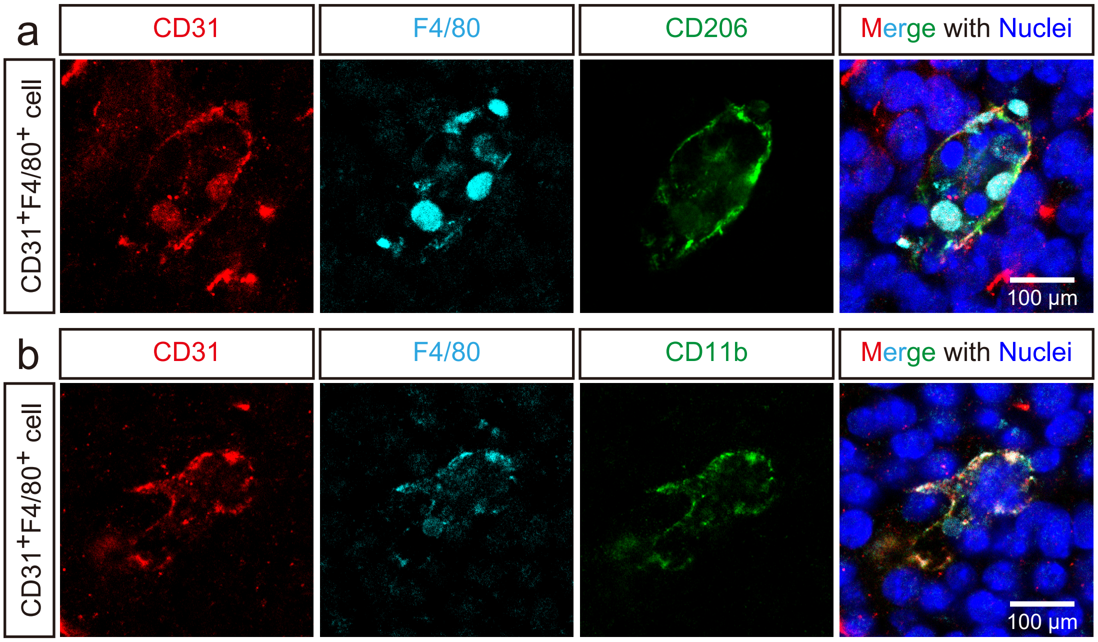

Supplementary Figure 3. CD31$^+$F4/80$^+$ cells express multiple macrophage surface markers.

CD31$^+$F4/80$^+$ cells, which infiltrate the dorsal midline at E10.5, express multiple macrophage markers. (a) CD31$^+$F4/80$^+$ cells express CD206, an M2 macrophage marker. (b) CD31$^+$F4/80$^+$ cells express CD11b, a pan-marker for monocytes/macrophages. CD31 (red), F4/80 (cyan), CD206/CD11b (green), and nuclei (blue).

Supplementary Figure 4

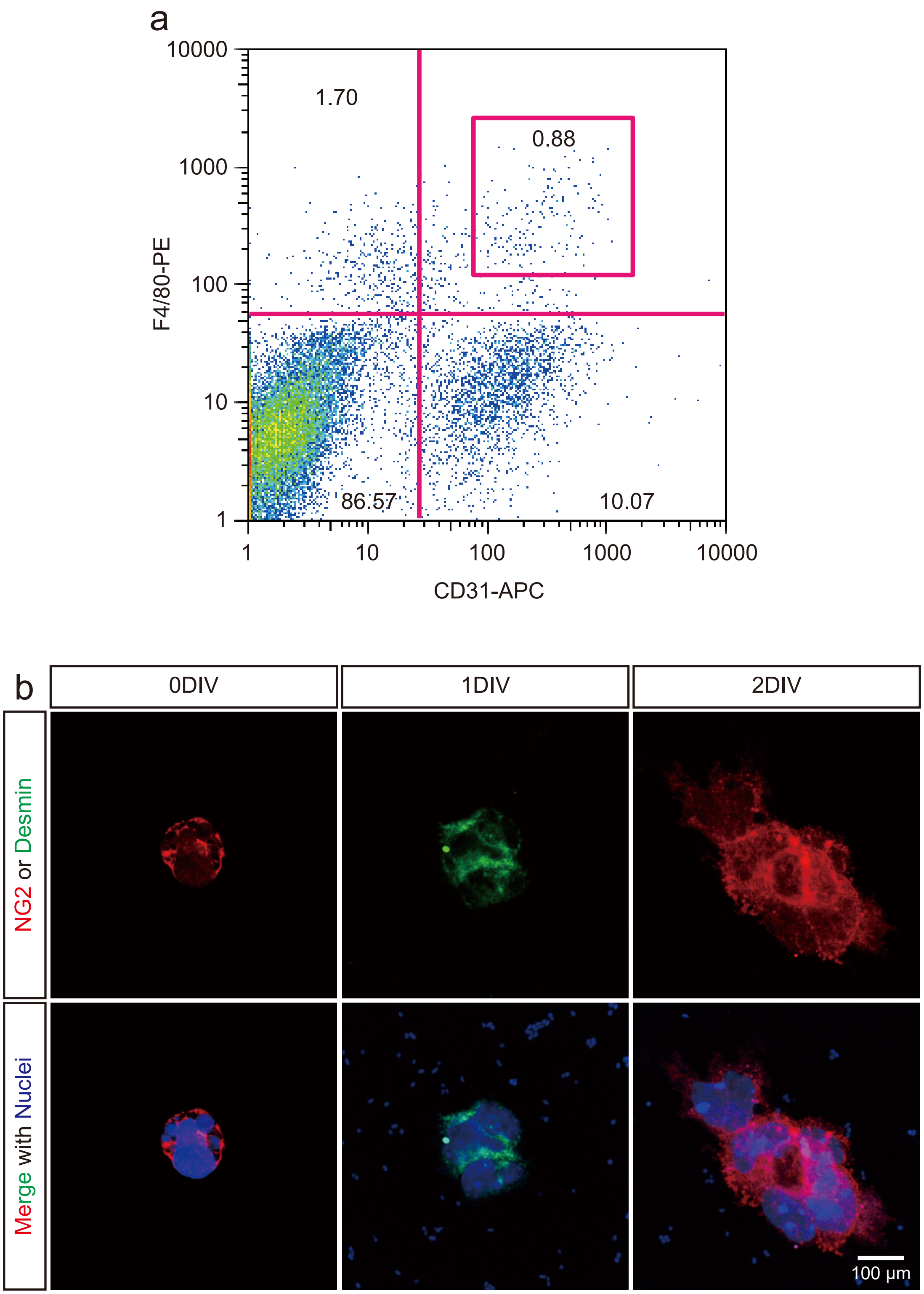

Supplementary Figure 4. Fractionation of phagocytes by flow cytometry using their CD31 and F4/80 surface markers and subsequent *in vitro* culture.

(a) $CD31^+F4/80^+$ cells fractionated from the E10.5 dorsal midline area by flow cytometry. The double positive fraction is less than 1%. (b) Cultured $CD31^+F4/80^+$ cells express the pericyte markers NG2 (red) and desmin (green). On 0DIV, fractionated $CD31^+F4/80^+$ cells exhibit a phagocytic morphology. On 1DIV, the $CD31^+F4/80^+$ cells proliferate and express desmin (green). On 2DIV, a couple of daughter cells migrate and express NG2 (red). Nuclei are counterstained with TO-PRO-3 (blue).

Supplementary Figure 5

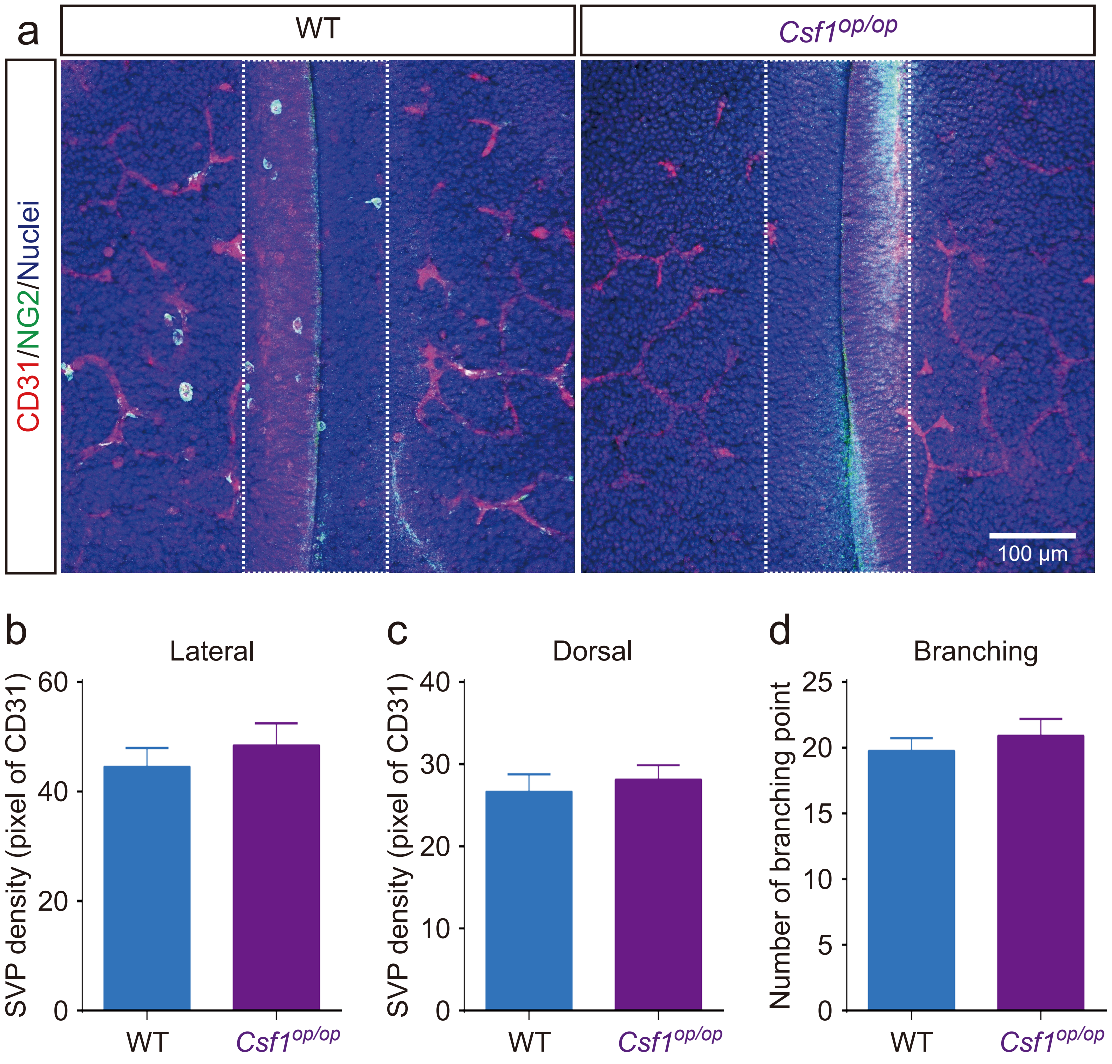

Supplementary Figure 5. E10.5 *Csf1*$^{op/op}$ mouse embryos do not display NG2^{+} cells at the dorsal midline area.

(a) In WT littermates, NG2^{+} cells (green, equivalent to the CD31^{+}F4/80^{+} cells) are observed in the dorsal midline area (dotted area) at E10.5. In contrast, no NG2^{+} cells are observed in the *Csf1*$^{op/op}$ mouse embryos. Nuclei are counterstained with Hoechst (blue). (b-d) CD31^{+}F4/80^{+} cells may not be involved in SVP angiogenesis. There are no significant difference in SVP density on the lateral side (b) or dorsal side (c), or SVP branching point frequency (d) between the two genotypes (WT = 8; *Csf1*$^{op/op}$ = 7; 200 × 200 μm). All error bars indicate the mean ± s.e.m.

Supplementary Figure 6

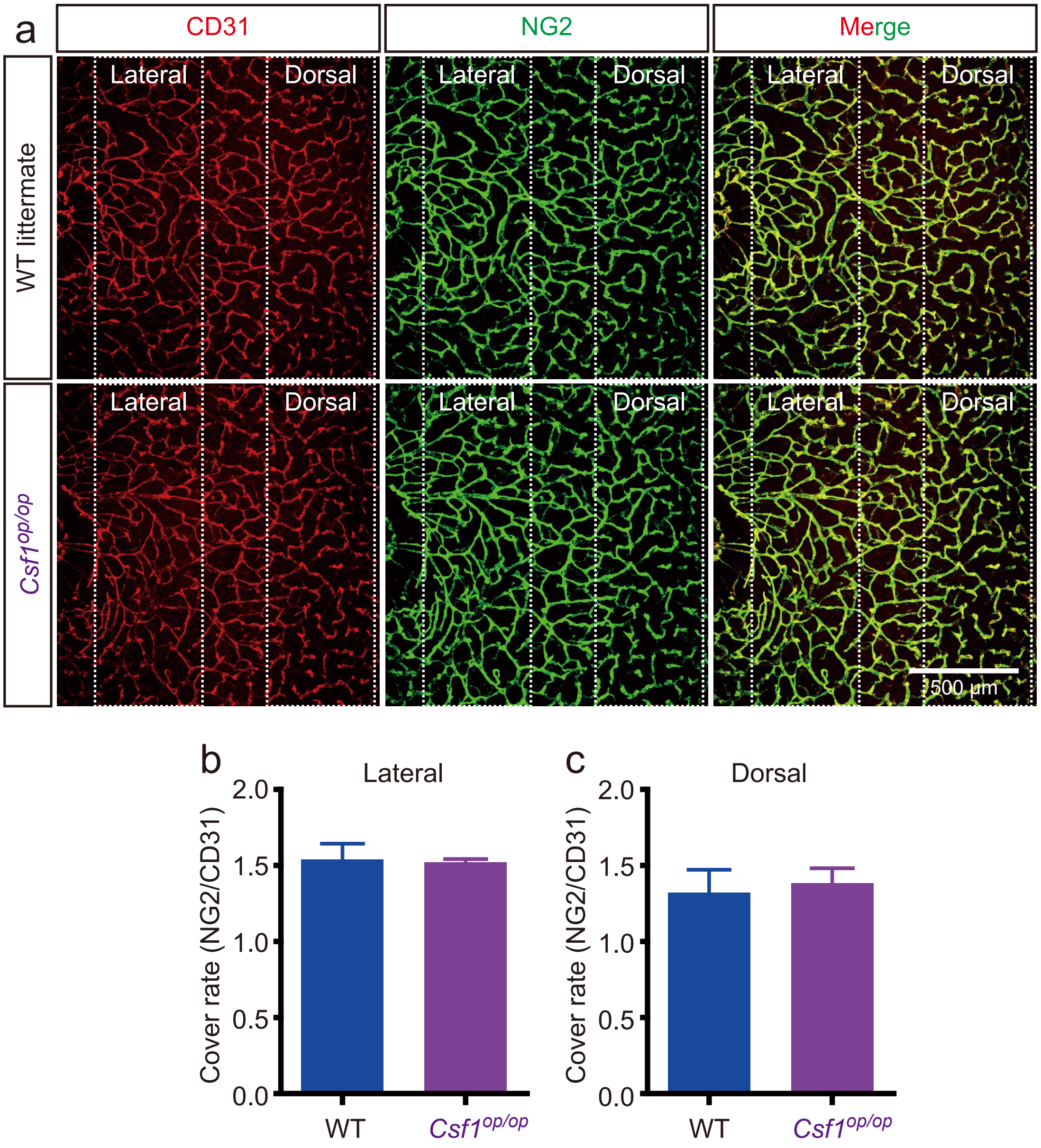

Supplementary Figure 6. E12.5 *Csf1$^{op/op}$* embryos show no change in pericyte coverage of the SVP.

(a) There is no significant difference in $NG2^{+}$ pericyte coverage in the dorsal midline area on E12.5 between *Csf1$^{op/op}$* mice and WT littermates. Statistical analysis further confirms no significant difference in the lateral (b) or dorsal (c) areas (ns, WT = 5, *Csf1$^{op/op}$* = 5, 400 × 400 μm). All error bars indicate the mean ± s.e.m.

Supplementary Figure 7

Gene Symbol	OP/WT ratio	Entrez Gene
630663	23.033	107849
Hand1	6.171	15110
Fgf8	5.667	14179
Fgf2	4.650	14173
Shh	4.543	20423
Gbx2	3.715	14472
Prl7d1	3.707	18814
Thy1	3.556	21838
Prl2c2	3.466	18811
Fgf18	2.931	14172
Sema5a	2.068	20356
Angpt2	1.768	11601
Scg2	1.766	20254
Adra2b	1.537	11552
Runx1	1.531	12394
Wisp3	1.526	327743
Serpinf1	1.510	20317
Foxc2	1.498	14234
Cyr61	1.486	16007
Cx3cl1	1.401	20312
Shc1	1.375	20416
Col4a3	1.321	12828
Lect1	1.313	16840
Tbx1	1.310	21380
Pitx2	1.305	18741
Tbx20	1.293	57246
Bmp4	1.271	12159
Thbs1	1.269	21825
Eng	1.254	13805
Il1b	1.253	16176
Tie1	1.234	21846
Meox2	1.228	17286
Ctgf	1.214	14219
Klf5	1.214	12224
Nos3	1.214	18127
Pnpla6	1.212	50767
LOC640441	1.209	21825
Gna13	1.204	14674
Fgf9	1.193	14180
Epas1	1.192	100048537
C1galt1	1.189	94192
Id1	1.163	15901
Meis1	1.152	17268
B4galt1	1.151	14595
Sox18	1.147	20672
Ednra	1.147	13617
Vhl	1.130	22346
Notch4	1.126	18132
S1pr1	1.124	13609
Ubp1	1.114	22221
Amot	1.107	27494
Nrp1	1.107	18186
Hbegf	1.102	15200
Edn1	1.085	13614
Wasf2	1.059	242687
Erap1	1.043	80898
Col18a1	1.042	12822
Cxcr4	1.037	12767
Casp8	1.035	12370
Ptk2	1.030	14083
Nf1	1.028	18015
Elk3	1.016	13713
Vegfa	1.011	22339

Gene Symbol	OP/WT ratio	Entrez Gene
Acvrl1	0.999	11482
Hs6st1	0.998	100047260
Fgfr1	0.995	14182
Rbm15	0.994	229700
Tgfa	0.993	21802
Sox17	0.990	20671
Pten	0.985	19211
Map3k7	0.985	26409
Adamts1	0.984	11504
Dicer1	0.984	192119
Hif1a	0.984	15251
Mapk7	0.980	23939
Erbb2	0.979	13866
Pofut1	0.976	140484
Rhob	0.971	11852
Vezf1	0.971	22344
Flt1	0.969	14254
Pml	0.967	18854
Cxcl12	0.967	20315
Rtn4	0.959	68585
Acvr1	0.958	11477
Gpx1	0.956	14775
Pdgfa	0.952	18590
Rbpj	0.950	19664
Dll4	0.948	54485
Crhr2	0.945	12922
Epas1	0.935	13819
Vash1	0.930	238328
Pknox1	0.911	18771
Plxnd1	0.909	67784
Anxa2	0.905	12306
Plcd1	0.901	18799
Tgfbr2	0.896	21813
Bai1	0.891	107831
Hhex	0.890	15242
Ctnnb1	0.877	12387
Gata2	0.860	14461
Mapk14	0.856	26416
Fgfr2	0.826	14183
Tek	0.818	21687
Htatip2	0.802	53415
Plcd3	0.799	72469
Ihh	0.765	16147
Notch1	0.759	18128
Il18	0.667	16173
Cx3cr1	0.478	100047704
Ang	0.381	11727
Plg	0.367	18815
Ovol2	0.278	107586
Enpep	0.207	13809
Serpine1	0.195	18787
Tbx4	0.126	21387
Il1a	0.119	16175

Supplementary Figure 7. Microarray data regarding angiogenesis-related genes.

Microarray data showing the ratio of angiogenesis-related genes expressed in the dorsal midline area of the *Csf1*$^{op/op}$ mouse embryo as compared with the WT littermate. Some genes are down-regulated; however, the expression levels of angiogenesis-related genes basically appear of a similar level to the WT littermate.

Supplementary Figure 8

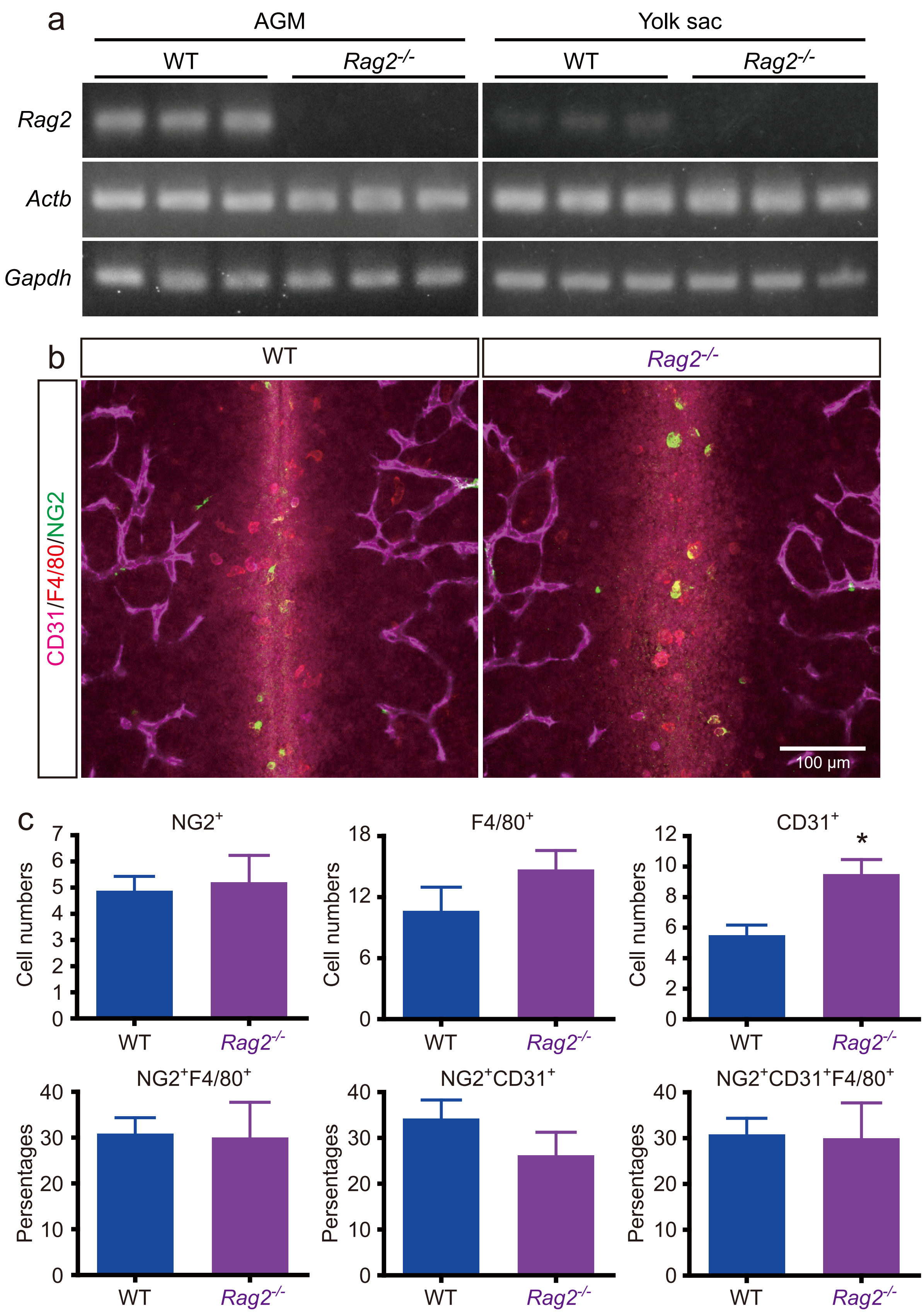

Supplementary Figure 8. *Rag2*$^{-/-}$ mice exhibit no alteration in NG2^{+} cell recruitment to the dorsal midline area.

(a) AGM and yolk sac *Rag2* mRNA expression levels are confirmed by RT-PCR. (b) Immunofluorescence on E10.5 *Rag2*$^{-/-}$ mouse embryos. Compared to their WT littermates, *Rag2*$^{-/-}$ mice showed no significant difference in NG2^{+} cells (green, equivalent to the CD31^{+}F4/80^{+} cells). (c) Statistical analysis of the cells infiltrating the dorsal midline area (WT = 4, *Rag2*$^{-/-}$ = 5, 200 × 200 μm). Cell surface marker analysis-based immunostaining shows essentially no difference between the 2 genotypes, with the exception of the single positive CD31^{+} cell population (*, $P < 0.05$). All error bars indicate the mean ± s.e.m.

Supplementary Figure 9

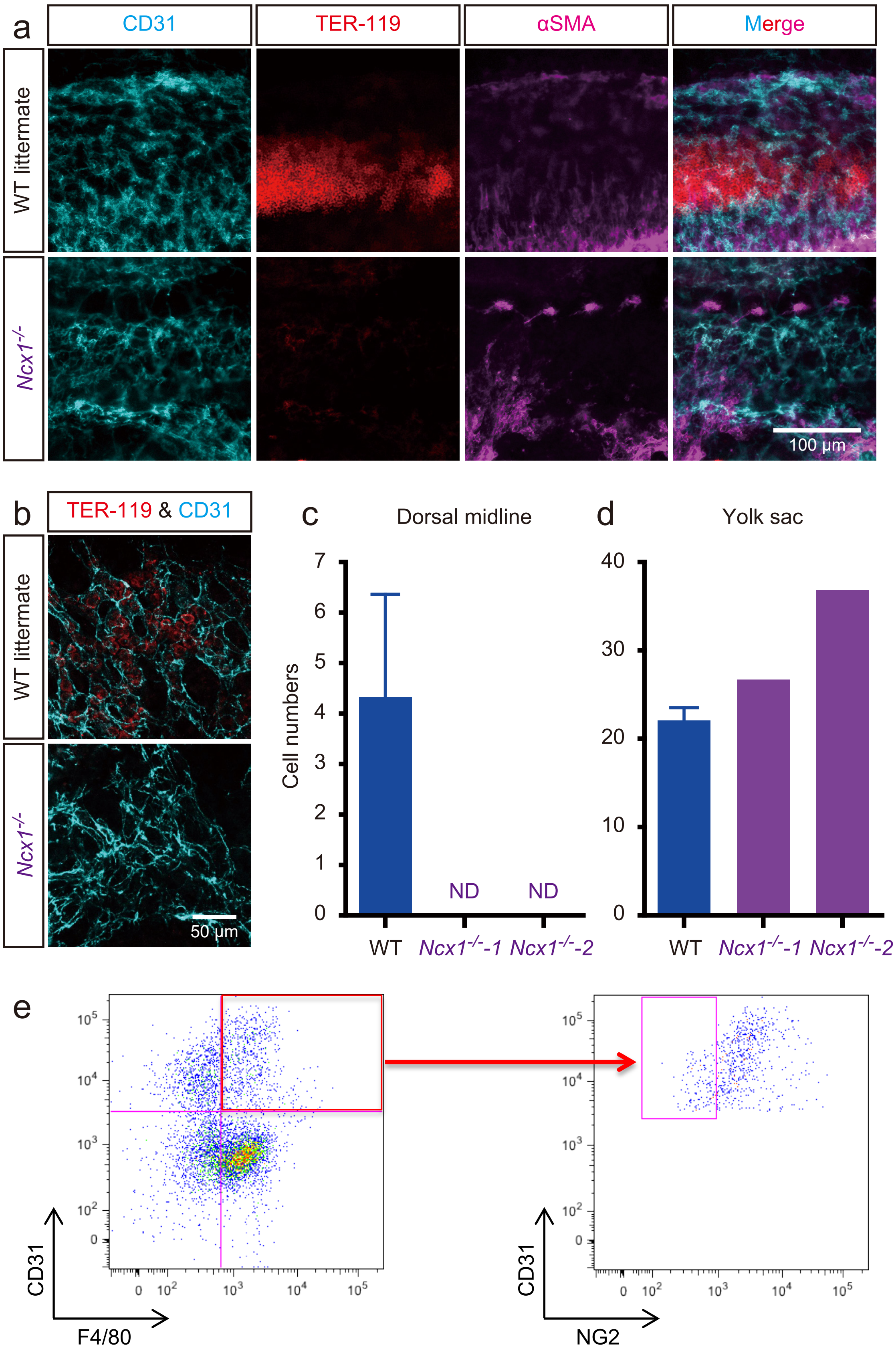

Supplementary Figure 9. NG2^{+} cells are not observed in the dorsal midline of *Ncx1*$^{-/-}$ mice and CD31^{+}F4/80^{+}NG2^{-} cells can be fractionated from yolk sac.

(a) Although TER-119^{+} embryonic erythrocytes (red) are recruited to the dorsal aorta region (cyan) encircled by αSMA^{+} cells (magenta) in the WT littermates, this event is not observed in *Ncx1*$^{-/-}$ mouse embryos on E10.5. (b) Compared to the embryos of WT littermates, *Ncx1*$^{-/-}$ mouse embryos also show no TER-119^{+} embryonic erythrocytes (red) in the cutaneous microvasculature (cyan). (c) In *Ncx1*$^{-/-}$ mice, no recruitment of the NG2^{+} cells (equivalent to the CD31^{+}F4/80^{+} cells) can be observed in the dorsal midline (WT = 3, *Ncx1*$^{-/-}$ = 2, 200 × 200 μm). (d) In contrast, yolk sac hematopoiesis, as surveyed by the major hematopoietic population, shows relatively increased TER-119^{+} erythrocyte numbers (WT = 3, *Ncx1*$^{-/-}$ = 2, 50 × 50 μm). (e) CD31^{+}F4/80^{+}NG2^{-} cells are fractionated from yolk sacs of mCherry^{+} embryos by flow cytometry.

Supplementary Figure 10

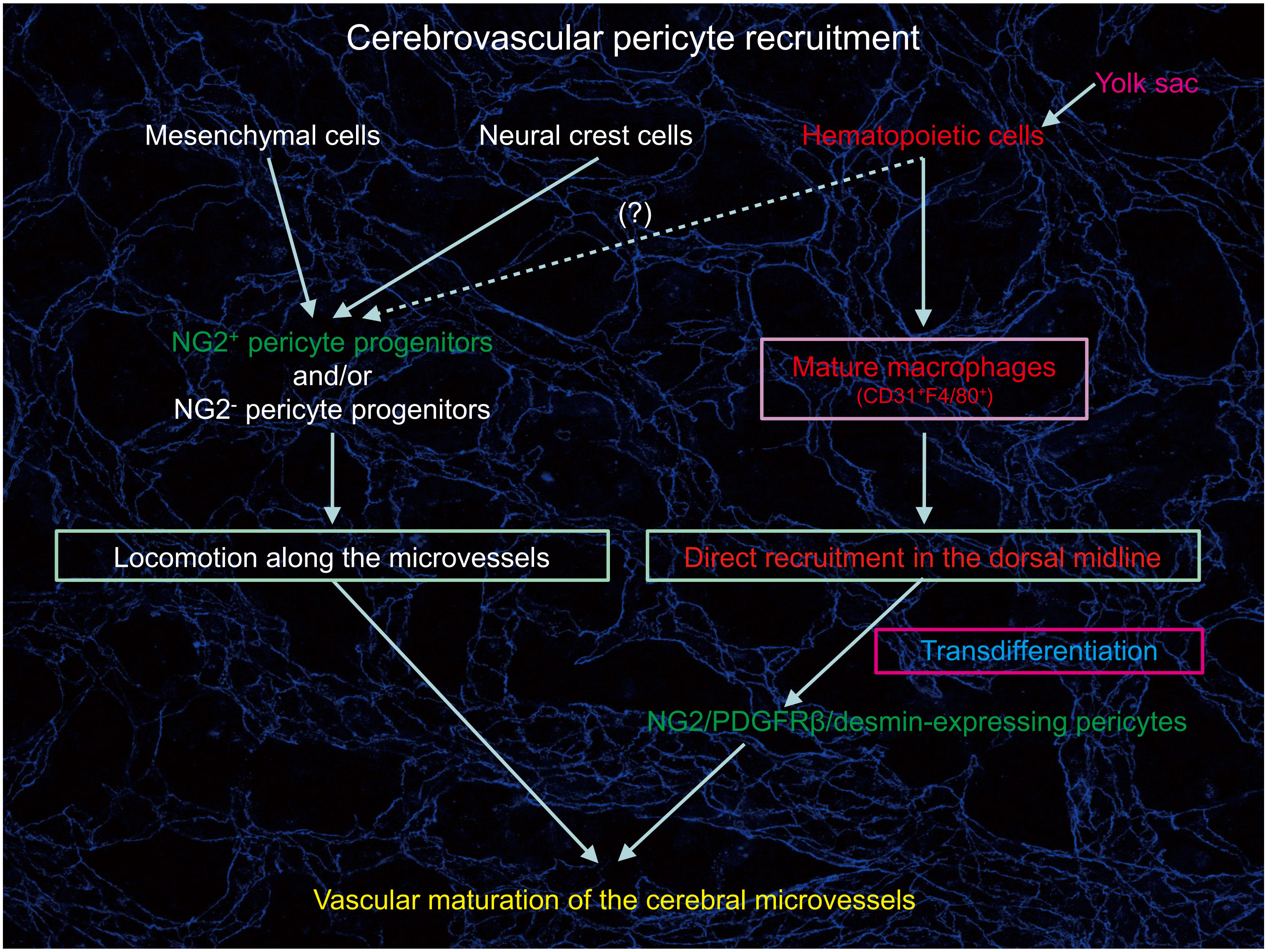

Supplementary Figure 10. Two cerebrovascular pericyte recruitment mechanisms. Direct recruitment to the dorsal midline (right scheme), which was demonstrated in this study *ad initium*, and locomotion along the microvessels (left scheme), which is well established in the vascular biology field, are suggested as two methods of pericyte recruitment.

Supplementary Movie 1

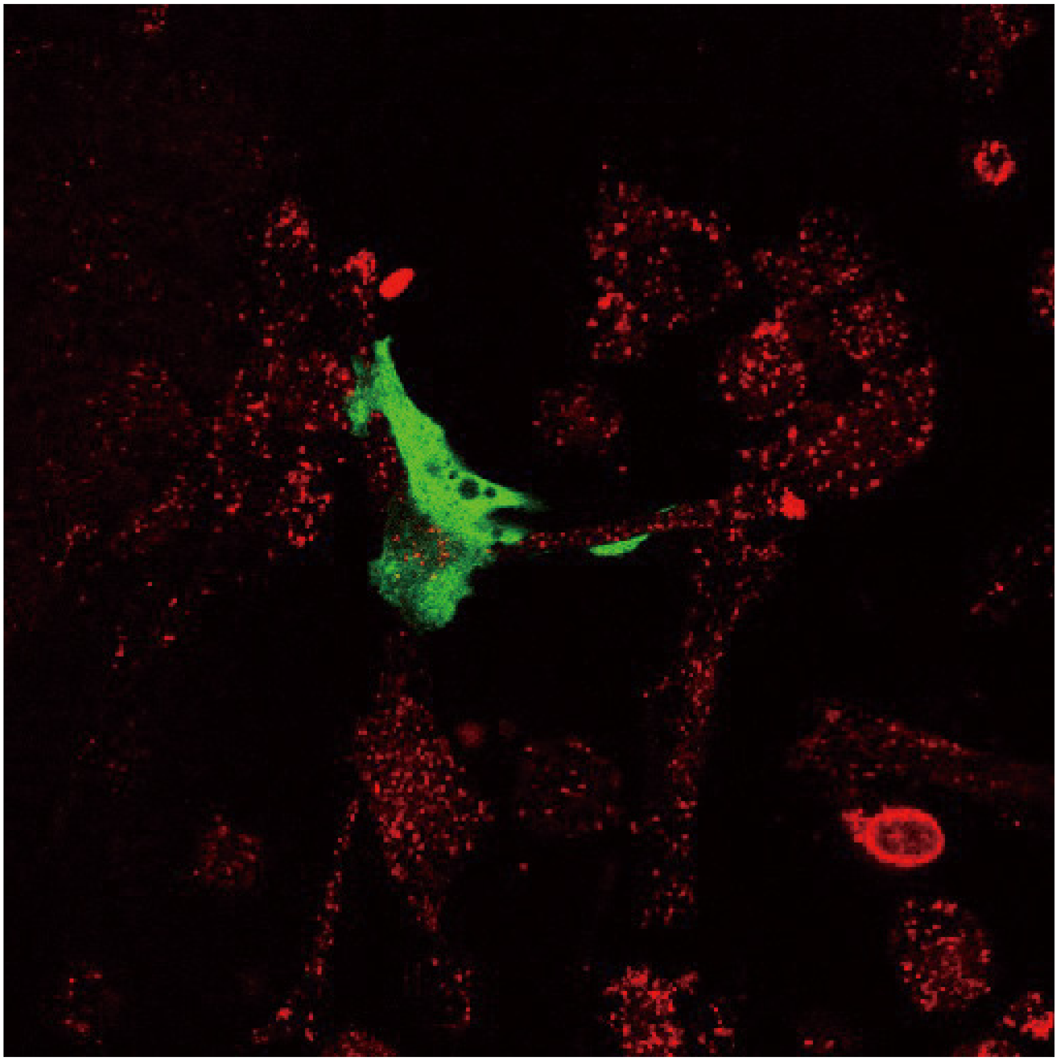

Supplementary Movie 1. Single-cell-tracing time-lapse analysis of target cell transdifferentiation.

The single-cell-tracing time-lapse study shows that EGFP reporter cells proliferate and transdifferentiate to pericytes *in vitro*.

Supplementary References

1. Bondjers, C. *et al.* Microarray analysis of blood microvessels from PDGF-B and PDGF-Rbeta mutant mice identifies novel markers for brain pericytes. *FASEB J* **20**, 1703-1705 (2006).
2. Cho, H., Kozasa, T., Bondjers, C., Betsholtz, C. & Kehrl, J. H. Pericyte-specific expression of Rgs5: implications for PDGF and EDG receptor signaling during vascular maturation. *FASEB J* **17**, 440-442 (2003).
3. Berger, M., Bergers, G., Arnold, B., Hammerling, G. J. & Ganss, R. Regulator of G-protein signaling-5 induction in pericytes coincides with active vessel remodeling during neovascularization. *Blood* **105**, 1094-1101 (2005).
4. Funk, J. L. *et al.* Expression of PTHrP and its cognate receptor in the rheumatoid synovial microcirculation. *Biochem Biophys Res Commun* **297**, 890-897 (2002).
5. Mazurais, D. *et al.* Cell type-specific localization of human cardiac S1P receptors. *J Histochem Cytochem* **50**, 661-670 (2002).
6. Takagi, H., King, G. L., Robinson, G. S., Ferrara, N. & Aiello, L. P. Adenosine mediates hypoxic induction of vascular endothelial growth factor in retinal pericytes and endothelial cells. *Invest Ophthalmol Vis Sci* **37**, 2165-2176 (1996).
7. Yonekura, H. *et al.* Placenta growth factor and vascular endothelial growth factor B and C expression in microvascular endothelial cells and pericytes. Implication in autocrine and paracrine regulation of angiogenesis. *J Biol Chem* **274**, 35172-35178 (1999).
8. Ikedo, H. *et al.* Smad protein and TGF-beta signaling in vascular smooth muscle cells. *Int J Mol Med* **11**, 645-650 (2003).
9. Fisher, S. A. & Ikebe, M. Developmental and tissue distribution of expression of nonmuscle and smooth muscle isoforms of myosin light chain kinase. *Biochem Biophys Res Commun* **217**, 696-703 (1995).
10. Apte, S. S., Fukai, N., Beier, D. R. & Olsen, B. R. The matrix metalloproteinase-14 (MMP-14) gene is structurally distinct from other MMP genes and is co-expressed with the TIMP-2 gene during mouse embryogenesis. *J Biol Chem* **272**, 25511-25517 (1997).
11. Frid, M. G., Shekhonin, B. V., Koteliansky, V. E. & Glukhova, M. A. Phenotypic changes of human smooth muscle cells during development: late expression of heavy caldesmon and calponin. *Dev Biol* **153**, 185-193 (1992).
